# Dose of nursing intervention: repercussions for professional practice

**DOI:** 10.1590/1980-220X-REEUSP-2025-0038en

**Published:** 2025-10-03

**Authors:** George Luiz Alves Santos, Thiago Augusto Soares Monteiro da Silva

**Affiliations:** 1 Centro Universitário La Salle, Niterói, RJ, Brazil.; 2 Universidade Federal do Rio de Janeiro, Escola de Enfermagem Anna Nery, Rio de Janeiro, RJ, Brazil.; 3 Universidade de Vassouras, Vassouras, RJ, Brazil.

**Keywords:** Nursing Process, Clinical Reasoning, Patient Care Planning, Dosage

## Abstract

This study aimed to understand the repercussions of the dose of nursing intervention for professional practice. This is a theoretical reflection study that sought to answer: what are the repercussions of the dose of nursing intervention for professional practice? To answer this question, a *corpus* of selected texts was considered that allowed us to understand such repercussions. The following repercussions were identified: the possibility of measuring the efficacy and effectiveness of treatments prescribed by nurses; the reduction of variability in prescribed actions; the construction of a more solid evidence base so that nurses, in different contexts, feel confident in taking responsibility for clinical outcomes in nursing; the greater replicability of actions in nursing intervention studies; the increased potential for replicability of treatments prescribed in similar situations, among others. The study’s objective was achieved as it identified how the dose of nursing intervention affects professional practice, without which it is not possible to discuss the impacts of care on patients’ clinical outcomes.

## INTRODUCTION

Planning care requires that nurses consider, essentially, three elements of nursing care, namely nursing diagnoses, outcomes and interventions. These elements can be identified in the current resolution, which deals with the Nursing Process (NP) implementation^(1)^. Nursing planning refers to the “development of a care plan directed at the person, family, community, special groups, and shared with the subjects of care and the nursing and health team”^(1)^. In order to plan in light of elements of nursing care, it is necessary to prioritize specific nursing diagnoses, as they will be essential for selecting the most appropriate clinical treatment. In cases where feasible, the therapeutic proposal—cure, rehabilitation, comfort, prevention, promotion, or even daily care rearrangement—ought to be delineated in collaboration with patients, in the presence of partial or total dependence, as well as the anticipated outcomes. In this regard, the selection of the most accurate nursing care elements presupposes considering diagnostic and therapeutic decision-making, a critical perspective that is also appropriate when considering the assessment of expected and feasible nursing and health outcomes^(1)^.

In the context of the health team, each member needs to elucidate the contributing aspects that delimit their disciplinary know-how, better understood when considering a multidisciplinary therapeutic plan, of which the nursing care plan is a part. In the meantime, the contribution of each member will be demonstrated based on specialized courses of action, decided and agreed upon, whenever possible, with care participants, ideally in a joint planning exercise, evidencing individual and collective therapeutic decision-making. “Therapeutic decisions, in the process of solving clinical problems, involve the idealization of plans or courses of action that aim to change the current situation of the problem to a better one”^(2)^, whenever possible.

“Each health profession has a way of describing “what” it knows”^(3)^ and, in the case of nursing in particular, according to its epistemology, what it knows concerns nursing diagnoses. Due to their characteristics, and in light of the standardized language system considered in this theoretical reflection, are classified into problem-focused, risk, and health promotion nursing diagnoses^(3)^. In this regard, it is argued that nurses treat “a human response to health problems and/or life processes, or a susceptibility to such a response”^(3)^. It is also worth noting that their knowledge must be guided by theoretical frameworks specific to nursing, fundamentally so that health and nursing practices are recognized, unequivocally, as being those of nurses, outlining nursing’s clinical-care contribution in the various socio-environmental contexts; only then can the nursing episteme be established.

In addition to discussions about “what” it knows – nursing diagnosis – or even about “how” it acts in relation to what it knows^(3)^ – nursing intervention –, as a scientific discipline of health sciences, its representatives must specifically clarify how much they act in relation to this “how”. They must inform in their nursing care plans the dose of nursing intervention necessary to treat a specific human response identified as requiring professional intervention, considering the three types of possible presentations considered in this text, in light of the standardized language system in particular, such as problem-focused, risk, and health promotion nursing diagnoses^(3)^.

This theoretical reflection is concerned with understanding aspects related to how nurses act based on the selection of a specific nursing intervention, but the context in which such intervention is circumscribed cannot be disregarded –NP implementation, which includes, in addition to the interventions, other stages, which is why the considerations throughout the text considered it. To elucidate “how” they act, it is necessary to define what a nursing intervention is, since in the planning stage addressed in the current resolution on the NP, there is talk of “therapeutic decision-making”^(1)^, which presupposes adopting a certain course of action. Nursing interventions are understood as “any treatment, based upon clinical judgment and knowledge, that a nurse performs to enhance patient/client outcomes. Nursing interventions encompass direct and indirect care”^(4)^.

The relevance of this topic lies in the scarcity of studies that address the issue of dose of nursing intervention in depth, as “it is important to determine the dose of an intervention to establish its effect in practice”^(4)^, however “at present, there is no solution to the problem of intervention dose”^(4)^. Counting the time needed to carry out an intervention, counting the number and extent of activities selected and implemented in an intervention have been pointed out as potential solutions to this theoretical-practical impasse^(4)^.

In the literature, two theoretical perspectives are identified to understand the dosage issue: the care perspective and the nursing personnel management perspective. From the first perspective, the term “dose of nursing intervention” is identified, whose essential attributes would be the amount, frequency, and duration of an intervention^(5)^. Regarding the practical implications of the topic, the authors state that “measuring and reporting the dose of nursing intervention in research is essential to the development of an evidence base adequate to support practice”^(5)^. Based on Sidani and Braden (1998), the authors^(5)^ define amount as the “quantity of nursing intervention delivered at one point in time”; frequency as the “number of times the intervention is delivered over a specified time period”; and duration as “the entire length of time over which the intervention is delivered”.

The perspective adopted by the authors to express the amount of interventions delivered in the study above^(5)^ was based on each intervention label from the Nursing Interventions Classification, not on the activities that comprised them – intervention units –, defined as “a set of activities carried out at the same time and related to a single intervention”^(5)^. The authors’ focus on the intervention label is intriguing, but it is understood that each label reflects the series of activities that comprise it. In view of this, reflecting on the activities means considering their determining role in selecting the dosage, as they influence the nursing intervention itself as they enable the selection of different doses of nursing intervention, according to each particular activity.

Research on dose of nursing intervention can help nurses in clinical practice to have clarity about “what nursing interventions to provide and how much of each intervention should be provided to achieve a desired outcome”^(5)^, minimizing variability in patient care. To this end, it is equally important to include discussions that address the nursing activities integrated into these interventions, since they structure them.

The second perspective, nursing staff management, can be found from the term “nurse dose”, defined as “the level (i.e., number and type) of nursing staff required to provide care that produces intended patient outcomes”^(6)^. In this context, for the authors, “nurse dose” would be “a structural variable capturing nurses’ capacity to deliver high quality care in acute care hospitals”^(6)^.

In light of the authors cited above, two attributes reflect the concept of nursing dose: active ingredients and intensity. The first would represent the essential and differentiating elements of nurses in relation to other healthcare professionals, and would have as critical indicators nurses’ theoretical and practical knowledge, and combination of skills. Intensity would represent the potential for nurse-patient interactions, being operationalized in terms of amount and frequency^(6)^. The perspective from which the reflections in this study will be carried out is close to the understanding of the term “dose of nursing intervention” in relation to nursing care and its repercussions^(5)^.

The literature has raised concerns about the dose of nursing intervention issue, “even more so if we consider the path to be taken in structuring the nursing clinic, in the development of solidified clinical research, monitoring the dose of nursing intervention is crucial. It requires demonstrating the efficacy and effectiveness of interventions, the specificities of the therapeutic dose used”^(7)^ in the face of a phenomenon identified as being treatable by nurses.

Considering the above, it is expected that this study will contribute to professional practice and assist in therapeutic decision-making, considering all prescriptive elements, namely: date of preparation of prescription; verb declaring the action to be performed; who should perform it: the subject; the descriptive phrase that includes how, when, where, how often, for how long or how much; in addition to the prescriber’s signature^(8)^. A review study pointed out difficulty in discerning the dose of the intervention in the reviewed studies to support replicability^(9)^. Discussing from the perspective of “for how long or how much?”^(8)^ can help to uncover more accurate and replicable therapeutic decision-making in nursing. Thus, the aim was to understand the repercussions of the dose of nursing intervention for professional practice.

## METHOD

This is a theoretical reflection study, developed from a *corpus* of texts selected for convenience that supported the understanding of the problem raised, i.e., the understanding of the repercussions of the dose of nursing intervention for professional practice. The reflective exercise began from the following question: what are the repercussions of the dose of nursing intervention for professional practice? It was considered that, in daily care, nursing activities are at the lowest level of abstraction and greatest concreteness, and are a comprehensive and inseparable part of nursing interventions, according to the standardized language system adopted in this reflection, i.e., the Nursing Interventions Classification^(4)^, starting from the activities to understand the repercussions of the intervention with regard to the dose of the nursing intervention. Nursing diagnoses were considered based on NANDA-International nursing diagnoses^(3)^.

Considering ethical issues, the study does not require submission to the Research Ethics Committee; however, access to research sources was ensured in an ethical manner, guaranteeing scientific integrity from them as well as good practices for the production of knowledge presented in this theoretical reflection. To this end, the reflective topic “Repercussions of the dose of nursing intervention for professional practice” was outlined.

### Repercussions of the Dose of Nursing Intervention for Professional Practice

To better contextualize the problem raised, a simulated clinical case is used, in the context of perioperative nursing, with pain as the phenomenon of interest:

A 67-year-old older woman underwent urgent, successful surgery to treat a femoral neck fracture on August 23, 2024, at approximately 11:00 p.m. On August 24, early in the morning, at 6:00 a.m., the patient informed the nurse on duty that she felt pain at the site where the surgical procedure was performed, while he was making clinical surveillance rounds of his patients. At the time, the patient was assessed using the Verbal Numeric Scale (VNS), reporting her pain level as 8 (0-10). Afterwards, the nurse reviewed the medical prescription and identified item 3 – dipyrone 1.0 g – intravenous (IV) 6/6 hours at 7:00 a.m. Once the measures related to safe practice for medication administration were taken, the prescribed item was administered, and the patient, approximately 35 minutes after being medicated, already reported a pain level of 4 points on the VNS, compared to the first assessment. After 60 minutes, she reported a slight discomfort, which was quantified at 1 point on the VNS, at the time of reassessment by the nurse.

Based on the simulated clinical case, it is important to highlight that dipyrone, depending on patients’ age, weight or even the route of administration, will have different dosages and different onset times of action: for adults and adolescents over 15 years of age (> 31 kg), doses between 500 and 750 mg are considered, orally 1 – 4 times/day, and doses between 1,000 and 2,500 mg, intramuscularly or intravenously, 1 – 4 times/day. In pediatrics (over 3 months), for instance, the oral dose for children weighing up to 30 kg should be between 10 and 15 mg/kg up to 4 times/day. Dipyrone is indicated as a treatment for painful manifestations and fever^(10)^.

It is understood, from the previous paragraph, that the collaborative intervention of analgesic administration^(4)^ is supported by clear prescriptive criteria (age group, patient weight and route of administration) for the medication (treatment) in question, i.e., minimum and maximum doses, in addition to the administration interval between doses, which were selected based on such criteria. Based on the clinical case in question, it is considered that nurses must similarly seek clear dosage criteria for nursing care so that these criteria allow them to intervene autonomously in phenomena of particular interest to nursing and to circumscribe, objectively, how to prescribe treatments based on nursing interventions and the dosages that they can encompass.

In light of disciplinary knowledge—the basis of autonomous nursing practice—nurses must be able to understand "what" nursing knows—nursing diagnoses—and "how" it treats this "what"—nursing interventions—as well as potential clinical outcomes—expected and achievable nursing and health outcomes^(1)^. To influence these outcomes, it is necessary to consider "how much" of the "how" is implemented, based on a specific "what"—the dose of nursing intervention. This would be the basis for improving expected and achievable outcomes^(1)^ and modifying clinical outcomes in nursing autonomously, uncovering the relationships among the nursing care elements within the nursing care plan. It should also be considered that nursing interventions are related in such a way as to modify health behaviors. Hence, it is worth reflecting: how to dose information in the context of health education?

Once a diagnosis is identified, an initial expected and achievable outcome^(1)^ is established, a “initial picture”^1^ of the reality to be addressed. With this “initial picture” in hand, nurses use nursing interventions to address the priority nursing diagnosis or “work diagnosis”^2^, in order to impact potential clinical outcomes. From the reassessments, there will be “partial photos” and/or “final photos” – patient responses to the prescribed and implemented treatments. “Work diagnosis”, in this reflection, can be understood as the nursing phenomenon to be addressed in a given time frame in the nursing care plan; for instance, over the course of 12 or 24 hours of a shift, despite other diagnostic possibilities.

It is based on the “work diagnosis” that nurses will analyze and establish how patients behaved in light of nursing care plan that was planned and implemented, allowing short, medium and long-term clinical outcomes to be identified and monitored – the basis for nursing evolution. It is important to keep in mind that the initial expected and feasible outcomes^(1)^ come from the identified human response which, depending on its type, must be improved, reduced, maintained or otherwise classified as appropriate. In view of this, initial outcomes, based on an evaluative time frame, will gain the connotation of partial or final when re-assess, an action characteristic of nursing evolution.

The identification of “existing problems, vulnerability conditions or dispositions to improve health behaviors”^(1)^ will be the basis for therapeutic decision-making in nursing. Existing problems and/or vulnerability conditions may express different degrees of severity, such as the extent of presentation of the identified phenomenon, challenging nurses to select the dose of nursing intervention according to the severity expressed by each patient, because the treatment and dose selected and prescribed, taking the identified phenomenon as a reference, must have the capacity to reverse, stabilize and/or maintain a given prioritized clinical condition – a nursing diagnosis. The perspective of a status for the nursing diagnosis is anchored in nursing literature^(7,11)^.

Also, from the perspective of healthcare risk management, nursing interventions and, as a consequence, the doses selected and prescribed, must be capable of adequately managing identified clinical risks. Suppose a given patient is admitted to a hospital unit and, based on a risk assessment instrument^(12)^, a nurse identifies that he has a high risk of falls, as he achieved a score of 14 points in the assessment, then, in the next moment, another patient is admitted, who scored 10 points, and was classified as having a moderate risk of falls, the nurse responsible for the two nursing care plans selects the intervention prevention against falls^(4)^, defined as “institution of special precautions in a patient at risk of injury due to a fall”^(4)^. It is understood, from the scores achieved, that although they represent similar diagnostic labels – risk of falls in adults^(3)^ –, the severity of the phenomenon was different, justified by different scores and, therefore, will require doses of nursing intervention considering different “dosages”.

Expanding on the reflections based on the previous simulated clinical situations, in the first case, we are dealing with a 70-year-old patient with a higher level of education, a retired accountant, but not very active and dependent for self-care. Furthermore, he demonstrated some resistance to the guidelines and denies the risk. In the second example, we are dealing with another older adult, now 75 years old, active, who, despite having less education, demonstrates greater adherence to preventive guidelines. Both patients have a diagnosis of risk of falls in adults^(3)^, but they express it clinically in different ways. Based on this provocation, the question is: what dose of nursing intervention should be prescribed in the form of guidance and clinical monitoring for both patients, since the risk presents itself in different intensities or degrees? Although the socio-environmental context adopted in the examples above is the hospital, it is possible to extrapolate the reflections of this text to other scenarios, such as Primary Health Care, since it is the prioritized phenomenon, as well as its clinical characteristics, that will be mandatory for selecting the dose of nursing intervention.

Based on relevant literature^(1,3,4,5,6,7,11)^, it is understood that the dose of nursing intervention refers to the quantity or intensity necessary for a given nursing intervention, when implemented, to achieve the expected effect, in order to treat a person, family, community or special groups that present a certain human response, influencing expected and feasible nursing and health outcomes, and modifying or maintaining clinical nursing outcomes by changing the status – present, improved, worsened, unchanged or resolved – of a given priority nursing diagnosis or work in a given socio-environmental context. Interventions that include quantifiable aspects, represented by milliliters (ml), milligrams (mg), centimeters (cm), among other measurements, should have their dose considered based on this type of parameter, objectively expressing the perspective dose of the intervention to be prescribed and implemented.

When nursing interventions involve behavioral and/or attitudinal aspects, having as a prescriptive basis the interaction between nurses and patients, it is inferred that the dose of nursing intervention is understood based on measures of interactive intensity and regularity, as they are closely related to the phenomenon of intervention provision repetition and/or reinforcement. For instance, those nursing interventions that include health education guidelines, since the dose of the intervention has to do with modifications or maintenance of health behaviors expressed by the person, family, community or special groups that may require reinforcement of these same guidelines.

The theoretical-practical approach to the phenomenon of dose of nursing intervention, from this perspective, is covered by a complex movement guided by the interaction between nurse and person, family, community or special groups, and contains within it a relational component of its own that, in order to be understood, demands an authentic face-to-face encounter, in which both parties involved have a voice and space for contributing speech. The challenge of establishing the dose of nursing intervention in light of interactive phenomenon of health guidance is understandable, since each person’s context and culture are sensitive perspectives for interpreting the world and the interactions that occur within it. Therefore, these are factors that must be considered in the selection, prescription and implementation of nursing actions, i.e., in therapeutic decision-making in nursing, which must also consider the dose of nursing intervention.

Thus, a unique relational and human nursing praxis is revealed in the context of health sciences, without which it is not possible to know the dose or even establish its posology, which is permeated, among other aspects, by mutual respect and recognition of beings in interaction, in a given socio-environmental context guided by educational practice. The intensity of each intervention will be assessed on site and at the time nurses are putting it into practice at the bedside or in any other setting, as feedback, the level of understanding of a given guideline or even the ability to reproduce what was guided will be mandatory to understand what dose of nursing intervention in the different interactive educational contexts should be considered.

Similar to any other treatment, it must be considered that nursing interventions, from their implementation, have the possibility of the emergence of side effects that will not always be noticeable “to the naked eye”, as nursing interventions involve a significant amount of interaction. Therefore, this topic must be discussed and stressed to its fullest extent so that both the dose and the results of its implementation are considered as a whole, in which side effects and/or adverse events are taken into account. One way to understand how nursing interventions affect the interactional context would be the feelings of well-being or otherwise resulting from the actions put into practice, based on nursing prescriptions. In this sense, verbal and non-verbal communication must be considered.

The delimitation of an unequivocal field of action as nursing, which unanimously characterizes what is defended as “nursing care”, may succumb to the excess of prescriptions that value surveillance actions and daily care routine, sometimes understood as nursing care embedded, in many care scenarios, in procedural aspects alone. Clinical surveillance is part of nursing care, but actions that consider clinical treatment as a fact-changer are mandatory in order to increasingly delimit the field of disciplinary professional action. Clinical surveillance is an attitudinal component of nursing interventions, but it should not be the exclusive basis of nursing treatments.

It is necessary to move beyond verbs such as “to monitor”, “to accompany” and “to observe”, which have a connotation of strict clinical vigilance, to verbs that assume a prescriptive/resolutive character, such as “to perform”, “to administer”, “to guide”, “to implement”, “to debride”, “to remove” and “to add”, which are closer to autonomous courses of action and know-how, transposing referral actions. In this sense, nursing theories can help to delimit the particular character of clinical phenomena, such as those of nursing; therefore, in addition to helping to declare what is unique to nursing, they include, in this same delimitation, how to deal with the particularization of how they define such phenomena as those of nursing.

The selection of the dose of nursing intervention is related to individual clinical expertise, being related to the ability to understand the severity of the identified nursing diagnosis, the socio-environmental context in which it occurs and the potential clinical outcomes expected in the short, medium and long term, but also to the therapeutic proposal in light of multidisciplinary care plan, since, depending on the patient’s profile, whether a clinical or surgical patient, in rehabilitation care, in chronic care or even in palliative care, will influence the dose of nursing intervention. The dose will vary according to the extent of the presentation of identified clinical phenomena.

In this reflection, the concept of intervention is supported by the Nursing Interventions Classification, i.e., “any **treatment** (our emphasis) that, based on clinical judgment and knowledge, a nurse puts into practice to improve patient outcomes”^(4)^. The expected and achievable outcomes^(1)^ will be improved as the prescribed and implemented treatment modifies the status of the “work” nursing diagnosis, expressed through modifications or maintenance of signs and symptoms of clinical importance to nursing. In this sense, it is essential to understand that nursing diagnoses can be present, improved, worsened, unchanged, and resolved^(11)^.

In nursing evolution, the 5^th^ stage of the NP, questions such as “how did the patient respond to treatment in the last 12 or 24 hours?” are guidelines for accurate assessment processes that allow for corrections in the nursing care plan, given a favorable or unfavorable clinical prognosis identified, i.e., the “prediction of the course of a disease and is expressed as the probability of a particular event occurring in the future. Predictions are based on defined groups of patients and the outcome may be different for each individual. However, knowledge of probable prognoses is useful in determining the best treatment. Prognostic factors are characteristics associated with the outcome in patients with the disease in question”^(13)^; in the case of nursing, how human responses behave in a given context and time to assess and define the prognosis.

It is understood that, in the nursing care plan, it is not enough to select, prescribe and implement nursing interventions; it is necessary to discuss “how much” of each intervention should be “administered” and in what period. Not infrequently, in nursing prescriptions in many health institutions, especially hospitals, the nursing prescription is operationalized by two main items: a phrase in the infinitive, which determines the activity to be performed (what will be done); and a time (frequency or interval of performance of a given action). However, nurses and their teams should guide their actions based on questions such as “how much nursing care should be provided and at what intensity?”.

Nursing activities are understood to be “at the concrete level of action, as they are the specific conducts or actions taken to implement an intervention and which help patients to progress towards the expected result”^(4)^. The activities that make up each intervention can be considered steps that, when put into practice, provide a path to implementing a given intervention. Although the literature suggests that research could use activities as intervention dose predictors, it is understood that counting a given number of activities as a basis for defining a given dose does not encompass the understanding of nursing dose discussed in this reflection.

Chart 1 presents the nursing intervention “Pressure injury prevention”^(4)^ and the activities that structure it - the basis for the reflective exercise on the dose of the nursing intervention that we intend to propose in this article. In each activity, we sought to understand the potential doses or not to be considered. In some activities, due to the way they are described, more than one activity is included in the text, which would make it difficult to select a precise dose. It is also understood that the Nursing Interventions Classification does not have this type of commitment – an excessive level of description that, in the authors’ understanding of this reflection, would not be the classification’s purpose. However, the reflective exercise carried out helps to reveal the problem that we want to discuss.

**Chart 1:** Theoretical reflections based on a nursing intervention

**Intervention - Pressure injury prevention** Definition: prevention of pressure injuries for an individual at high risk of developing them.	
**Activities**	**Critical-reflective exercise**
1. Use a recognized risk assessment tool to monitor the individual’s risk factors (e.g., Braden scale).	1. Risk assessment tools help to understand the severity of the risk and to define the intensity of the prevention to be prescribed/implemented. In this case, the dose would be related to the assessment interval and the clinical surveillance implemented.
2. Use skin temperature measurement methods to determine the risk of pressure injuries, according to the institution’s protocol.	2. Measurement methods help to understand the severity of the risk and to define the intensity of the prevention to be prescribed. In this sense, the assessment interval would be appropriate here, which would support the selection of the dose or preventive resources to be selected.
3. Encourage the individual not to smoke and to avoid alcohol use.	3. This activity concerns guidance actions for smoking cessation and alcohol use. They involve adherence to guidance measures and need to consider the level of knowledge and engagement in smoking cessation. The dose here is related to the amount/intensity of information to be shared, as well as the number of approaches considered. Understanding and modifying behavior from the first nursing consultation would be the basis for selecting the dose, reassessed in other meetings, as it is necessary to consider how much the individual will be willing to modify risk behaviors.
4. Document any previous incidences of pressure injury formation.	4. Documentation is part of and results from the initial and ongoing assessment, and enables an understanding of how nursing treatment was carried out at a previous time. It is not the dose of nursing intervention that should be considered here, but the quality of the initial assessment and the professional records resulting from it.
5. Document weight and weight changes.	5. Changes in weight can guide the intensity of preventive measures and, as a consequence, the dose of nursing intervention, since, depending on the degree of risk, a series of technologies and nursing actions should be considered.
6. Document skin status on admission and daily.	6. Initial assessment of the skin and its response to prescribed treatment can assist in the scheduling and selection of preventive measures and treatments. Thus, there is a direct correlation between the current state of the skin, its potential clinical risk for developing a given lesion and the expected clinical outcomes. However, the activity concerns professional registration.
7. Intensively monitor areas of redness.	7. Monitoring is related to clinical nursing surveillance and should be understood, in this reality, as a dose, related to the intensity with which monitoring is carried out, having a direct relationship with the time interval and the time spent on the assessment.
8. Remove excessive skin moisture resulting from perspiration, wound drainage, and fecal and urinary incontinence.	8. Several factors contribute to excessive humidity, which makes it difficult to establish a dose considering the nursing activity in question, since exposure to humidity can occur at different times and from different causes. It would be appropriate to discuss in the prescription the dose related to continuous monitoring or the individualization of each problem/action/dose.
9. Apply protective barriers, such as creams or moisture-absorbing materials, to remove excess moisture as appropriate.	9. In addition to moisture removal, it is important to consider how much barrier cream should be applied, as this is related to the dose of the nursing intervention. Furthermore, the activity also mentions absorbent materials that would have another perspective on dose, as linked to their absorption capacity and, consequently, hours of protection that the technology in question can offer due to the retention capacity of a given volume of urine.
10. Remove positioning every 1 to 2 hours.	10. Repositioning is one of the activities that sensitively qualifies the understanding of what constitutes a dose of nursing intervention, as it is identified that the dose is linked to the interval in which it occurs, i.e., every 1 to 2 hours, a “decubitus change dose” will be administered to prevent the appearance of pressure injuries which, among other factors, are related to how much local ischemia a given tissue may be experiencing.
11. Perform patient position changes carefully (avoid deformation by tangential forces) to prevent damage to fragile skin.	11. This activity indicates how to do it and is related to the activity “Change positioning every 1 to 2 hours”; therefore, it would not be appropriate to discuss the dose of nursing intervention in this item, but rather the quality with which a given care action is performed.
12. Place the position change guide at the bedside, as appropriate.	12. The script indication serves as a basis so that the patient does not repeat the position before the indicated time; therefore, there is no room to discuss the dose of nursing intervention in this item, since the strategy would serve professionals as a guide for the activity.
13. Inspect the skin over bony prominences and other pressure points at least once/day during repositioning.	13. Monitoring is related to the intensity of a potential risk identified in a patient; therefore, depending on the level of risk, the monitoring dose should be readjusted based on the skin assessment at each reassessment, i.e., the amount of monitoring should be prescribed. In the case of this activity, the relationship between action and dose has to do with the time interval in which the actions are performed.
14. Avoid massaging over bony prominences.	14. Guidance on good clinical practice that complements the way of intervening. There is no room for discussion on the dose of nursing intervention.
15. Position the patient with pillows to elevate pressure points in bed.	15. Guidance on good clinical practice that complements the way of intervening. There is no room for discussion about the dose of nursing intervention.
16. Keep sheets clean, dry, and wrinkle-free.	16. Guidance on good clinical practice that complements the way of intervening. There is no room for discussion about the dose of nursing intervention.
17. Make the bed with toe pleats.	17. Guidance on good clinical practice that complements the way of intervening. There is no room for discussion about the dose of nursing intervention.
18. Use specialized beds and mattresses as appropriate.	18. The use of specialized beds and surfaces should be selected based on the degree or score of risk identified from a given clinical assessment instrument and, in this sense, such specialized beds and mattresses will be selected according to such severity. This relates to the “amount” of technologies available.
19. Use bed equipment that protects the patient.	19. Guidance on good clinical practice that complements the way of intervening. There is no room for discussion about the dose of nursing intervention.
20. Avoid “donut” equipment in the sacral area.	20. Guidance on good clinical practice that complements the way of intervening. There is no room for discussion about the dose of nursing intervention.
21. Moisturize dry, intact skin.	21. Hydration is related to the occurrence of pressure injuries; therefore, it is important to discuss the dose of hydration to be prescribed. The characteristics of the selected product will be mandatory for selecting the dose of the nursing intervention.
22. Avoid hot water and use mild soap when bathing.	22. Guidance on good clinical practice that complements the way of intervening. There is no room for discussion about the dose of nursing intervention.
23. Monitor for sources of pressure and friction.	23. The monitoring dose will be related to the severity of the risk of pressure injuries. An interval between monitoring sessions may be related to the dose in the sense of how much monitoring will be implemented.
24. Apply heel and elbow protectors as appropriate.	24. The application of protectors is related to the degree of risk, since, depending on the type of risk, a given technology is selected that is able to protect the patient and their skin. Therefore, following the manufacturer’s instructions is important. In the case of creams and sprays, the amount dispensed during application should be considered as the dose of the nursing intervention. It is important to highlight that the selection of the amount of barrier cream or protector is related to the extent of the surface at risk.
25. Facilitate frequent, small changes in the patient’s position.	25. Small repositioning will be considered in light of the identified risk of pressure injury; therefore, the dose is related to the change intervals and the degree of risk. In this understanding, the dose of the nursing intervention and the position change interval are inseparable.
26. Provide a trapeze to assist with frequent changes in the patient’s position.	26. Guidance on good clinical practice that complements the way of intervening. There is no room for discussion about the dose of nursing intervention.
27. Monitor the individual’s mobility and activity.	27. Mobility and activity levels will be mandatory for the intensity of implementation of preventive measures and, therefore, guide the definition of the dose of nursing intervention with regard to patient repositioning.
28. Ensure adequate dietary intake, especially of protein, vitamins B and C, iron, and calories, using supplements as appropriate.	28. Collaborative activity that, in order to be implemented, must be discussed with nutritionists and dietitians. In this sense, the dose of the intervention must be discussed jointly, but closely monitored by nurses and the nursing team, as they are the professionals who commonly administer or offer food to patients.
29. Assist the individual in maintaining a healthy weight.	29. Maintaining a healthy weight is a complex activity that encompasses a series of activities, and weight monitoring is a measure that can be implemented quickly and independently, but it should not be the only one. Nurses need to work together through a multidisciplinary therapeutic plan to help individuals maintain an appropriate weight for the variables defined by scientific evidence. The dose of this activity is related to the amount of guidance that would be provided for an individual to lose weight.
30. Instruct the family or caregiver about signs of skin breakdown, as appropriate.	30. This activity is related to learning and engaging in pressure injury prevention behaviors established by all dosage involved. The intervention dose is linked to the amount of information shared, the intensity of the approach and the interval between the guidelines, i.e., the interval between the doses of information offered.

Source: Adapted from Nursing Interventions Classification^(4)^.

The previous reflective exercise revealed the complex but necessary discussion of the dose of nursing intervention at the level of concrete activities or actions, since they will be prescribed and implemented in care settings by nurses and team, and which will guide the care actions that they represent through nursing prescription. In this sense, indicating courses of action is indisputable, as is clearly describing to what extent these same actions should be carried out – how much and how. Treatments must necessarily consider doses to be prescribed.

In the context of nursing care, families and significant others are important therapeutic instruments that patients have at their disposal to continue treatments or even prevent health complications, acting as partners - fundamental pieces that should be incorporated at the time of selection and implementation of health and nursing actions. Thus, critically assessing how family members and significant others understand the nursing care plan is essential, since, when selecting nursing interventions and their respective doses, they should be considered.

The limitation of this research is in the context of its approach, i.e., a theoretical reflection, since the complexity of the problem discussed here could be considered by more robust methods. The research responded to the objective when, through the proposed reflective exercise, identified the potential repercussions of the dose of nursing intervention for professional practice.

## FINAL CONSIDERATIONS

To understand the dose of nursing intervention and its impact on professional practice, it is necessary to consider that its prescription must be based on clear dosage criteria. Without these criteria, it becomes unlikely, in daily care, to determine the intervention dose and/or measure the impacts of treatments prescribed by nurses on patients’ clinical outcomes. The perspective of the dose of nursing intervention presented in this text is delimited by the Nursing Interventions Classification. In essence, a set of activities makes up a nursing intervention. Understanding how each activity may require a specific dose, and how the integrated set of activities expresses the dose of an intervention as a whole, is fundamental to understanding its repercussions on professional nursing practice.

In light of the reflective exercise carried out and the selected text *corpus*, the repercussions outlined include: the possibility of measuring the efficacy and effectiveness of treatments prescribed by nurses; the reduction of variability in prescribed actions, as it will be possible not only to choose a course of action, but also to determine it, taking into account parameters such as frequency, intensity, and amount; greater clarity for nursing assistants and technicians about the practice standards established by nurses; the construction of a more solid evidence base so that nurses, in different contexts, feel confident in taking responsibility for clinical outcomes in nursing; greater replicability of interventions/activities in nursing intervention studies.

Furthermore, there is greater clarity on the delineation of autonomous actions of nurses as part of the health team and an increased potential for replicability of treatments prescribed in similar situations, which can contribute to the acquisition of clinical expertise in diagnostic or therapeutic decision-making processes. Finally, there is greater clarity for future updates to the Nursing Interventions Classification and better guidance on how to effectively prescribe care, impacting expected and achievable outcomes and the status of prioritized diagnoses in the context of clinical practice.

## Data Availability

The entire dataset supporting the results of this study was published in the article itself.

## Footnotes

1 The association of expected nursing outcomes with photographic records or “initial”, “partial” and “final” photos refers to the action of photographing wounds or injuries so that an initial parameter can be obtained and, from this, their evolution can be established in a given care context, according to an established period, allowing the assessment of the expected and achieved outcomes.

2 The expression “work diagnosis” was heard for the first time by the first author of this reflection, when he was talking to Professor Maria Márcia Bachion at an event celebrating the anniversary of the *Universidade Federal Fluminense Grupo de Estudos em Sistematização da Assistência de Enfermagem* (GESAE-UFF), held in Rio de Janeiro.
