# Supplementary material for: Dose of nursing intervention: repercussions for professional practice
Source: Rev Esc Enferm USP. 2025 Oct 3;59:e20250038. doi: 10.1590/1980-220X-REEUSP-2025-0038en (PMC12494363; doi:10.1590/1980-220X-REEUSP-2025-0038en)
Supplement: Translation

# Translation: Dose da intervenção de enfermagem: repercussões para a prática profissional

**DOI:** 10.1590/1980-220X-REEUSP-2025-0038pt

**Authors:** George Luiz Alves Santos, Thiago Augusto Soares Monteiro da Silva

**Affiliations:** 1 Centro Universitário La Salle, Niterói, RJ, Brasil.; 2 Universidade Federal do Rio de Janeiro, Escola de Enfermagem Anna Nery, Rio de Janeiro, RJ, Brasil.; 3 Universidade de Vassouras, Vassouras, RJ, Brasil.

**Keywords:** Processo de Enfermagem, Raciocínio Clínico, Plano Assistencial de Enfermagem, Posologia

## Abstract

Objetivou-se compreender as repercussões da dose da intervenção de enfermagem para a prática profissional. Trata-se de estudo de reflexão teórica, que procurou responder: quais são as repercussões da dose da intervenção de enfermagem para a prática profissional? Para responder a essa pergunta, foi considerado um *corpus* de textos selecionados que permitisse compreender tais repercussões. As seguintes repercussões foram identificadas: a possibilidade de mensurar a eficácia e efetividade dos tratamentos prescritos por enfermeiros; a redução da variabilidade nas ações prescritas; a construção de uma base mais sólida de evidências para que enfermeiros, em diversos contextos, se sintam seguros ao assumir a responsabilidade por desfechos clínicos em enfermagem; a maior replicabilidade das ações em estudos de intervenção de enfermagem; o aumento do potencial para replicabilidade de tratamentos prescritos em situações semelhantes, entre outras. O objetivo do estudo foi alcançado na medida em que identificou como a dose da intervenção de enfermagem repercute na prática profissional, sem a qual não há como se discutir os impactos assistenciais nos desfechos clínicos dos pacientes.

## INTRODUÇÃO

Planejar o assistir requer que os enfermeiros considerem, essencialmente, três elementos da assistência de enfermagem, quais sejam diagnósticos, resultados e intervenções de enfermagem. Tais elementos podem ser identificados na atual resolução, que trata da implementação do Processo de Enfermagem (PE)^(1)^. O planejamento de enfermagem refere-se ao “desenvolvimento de um plano assistencial direcionado para a pessoa, família, coletividade, grupos especiais, e compartilhado com os sujeitos do cuidado e equipe de enfermagem e saúde”^(1)^. Para planejar à luz dos elementos da assistência de enfermagem, devem-se priorizar determinados diagnósticos de enfermagem, que serão mandatórios para a seleção do tratamento clínico mais adequado, devendo-se, sempre que possível, em parceria com o cliente, definir a proposta terapêutica – cura, reabilitação, conforto, prevenção, promoção ou mesmo a reconfiguração de cuidados cotidianos – na vigência de dependência parcial ou total, bem como quais resultados estão prospectados. Nesse sentido, a seleção dos elementos de assistência de enfermagem mais acurados pressupõe considerar as tomadas de decisão diagnóstica e terapêutica, perspectiva crítica também adequada quando se considera a avaliação dos resultados esperados e exequíveis de enfermagem e de saúde^(1)^.

No contexto da equipe de saúde, cada integrante necessita elucidar os aspectos contributivos que delimitam seu saber-fazer disciplinar, melhor compreendidos quando se pensa em um plano terapêutico multidisciplinar, do qual o plano assistencial de enfermagem faz parte. Nesse ínterim, a contribuição de cada integrante será demonstrada a partir dos cursos de ação especializados, decididos e pactuados, sempre que possível, com o partícipe do cuidado, idealmente em um exercício de planificação conjunta, evidenciando a tomada de decisão terapêutica individual e coletiva. “As decisões terapêuticas, no processo de solução dos problemas clínicos, envolvem a idealização de planos ou cursos de ação que tenham por objetivos mudar a situação atual do problema para uma outra melhor”^(2)^, sempre que possível.

“Cada profissão na área da saúde tem uma maneira de descrever “o que” conhece”^(3)^ e, no caso da enfermagem em particular, recortado por sua epistemologia, o que ela conhece diz respeito aos diagnósticos de enfermagem. Estes, por suas características, e à luz do sistema de linguagem padronizada considerado nesta reflexão teórica, são classificados em diagnósticos de enfermagem com foco no problema, de risco ou de promoção da saúde^(3)^. Nesse sentido, advoga-se que enfermeiros tratam “uma resposta humana a problemas de saúde e/ou processos da vida, ou uma suscetibilidade a tal resposta”^(3)^. Destaca-se, ainda, que seus conhecimentos devem ser norteados por referenciais teóricos próprios da enfermagem, fundamentalmente para que as práticas de saúde e enfermagem sejam reconhecidas, de forma inequívoca, como sendo as dos enfermeiros, delineando-se a contribuição clínico-assistencial de enfermagem nos diversos contextos socioambientais; só assim a episteme de enfermagem poderá se sedimentar.

Para além das discussões sobre “o que” conhece – diagnóstico de enfermagem – ou ainda sobre “como” age em relação ao que conhece^(3)^ – intervenção de enfermagem–, enquanto disciplina científica das ciências da saúde, seus representantes devem elucidar especificamente o quanto agem em relação a esse “como”. Eles devem informar em seus planos assistenciais de enfermagem a dose de intervenção de enfermagem necessária para tratar uma determinada resposta humana identificada como necessitando de intervenção profissional, ponderando os três tipos de apresentações possíveis considerados neste texto, à luz do sistema de linguagem padronizada em particular, tais como diagnósticos de enfermagem com foco no problema, de risco ou de promoção da saúde^(3)^.

Esta reflexão teórica se ocupa em compreender aspectos relacionados ao quanto agem enfermeiros a partir da seleção de um determinado como – intervenção de enfermagem, mas não se pode desconsiderar o contexto no qual está circunscrita tal intervenção – a implementação do PE, que compreende, além das intervenções outras etapas, por isso as ponderações ao longo do texto consideraram-no. Para elucidar “como” agem, há que se definir o que seja uma intervenção de enfermagem, pois na etapa de planejamento de que trata a atual resolução sobre o PE, fala-se em “tomada de decisão terapêutica”^(1)^, que pressupõe adotar um determinado curso de ação. Intervenções de enfermagem são compreendidas como “qualquer tratamento que, baseado em julgamento e conhecimento clínico, um enfermeiro ponha em prática para melhorar os resultados do paciente. As intervenções de enfermagem incluem tanto os cuidados diretos quanto os indiretos”^(4)^.

A pertinência desta temática reside na escassez de estudos que abordem o tema dose da intervenção de enfermagem em profundidade, pois “é importante determinar a dose de uma intervenção para estabelecer o efeito dela na prática”^(4)^, contudo “no presente, não há solução para o problema da dose da intervenção”^(4)^. Têm sido apontadas como potenciais soluções para este impasse teórico-prático a contagem do tempo necessário à realização de uma intervenção, a contagem do número e a extensão de atividades selecionadas e implementadas de uma intervenção^(4)^.

Na literatura, identificam-se duas perspectivas teóricas para compreender a problemática da dosagem: a assistencial e a da gestão de pessoal de enfermagem. Sob a primeira perspectiva, identifica-se o termo “dose da intervenção de enfermagem”, cujos atributos essenciais seriam a quantidade, a frequência e a duração de uma intervenção^(5)^. Sobre a implicação prática da temática, os autores afirmam que “medir e relatar a dose da intervenção de enfermagem em pesquisa é essencial para o desenvolvimento de uma base de evidências adequada para dar suporte à prática”^(5)^. A partir de Sidani e Braden (1998), os autores^(5)^ definem quantidade como a “quantidade de intervenção de enfermagem entregue em um ponto no tempo”; frequência como o “número de vezes que a intervenção é entregue ao longo de um período de tempo especificado”; e duração como “o período de tempo inteiro durante o qual a intervenção é entregue”.

A perspectiva adotada pelos autores para expressar a quantidade de intervenções entregues no estudo acima^(5)^ deu-se a partir de cada rótulo de intervenção da Classificação das Intervenções de Enfermagem, não das atividades que as compunham – unidades de intervenção–, definidas como “um conjunto de atividades realizadas ao mesmo tempo e relacionadas a uma única intervenção”^(5)^. O enfoque dado pelos autores a partir do rótulo da intervenção é instigante, mas compreende-se que cada rótulo reflete a série de atividades que o integram. À vista disso, refletir sobre as atividades é considerar o papel determinante delas na seleção da dosagem, pois influenciam a própria intervenção de enfermagem à medida que possibilitam a seleção de diferentes doses de intervenção de enfermagem, conforme cada atividade em particular.

Pesquisar a respeito da dose da intervenção de enfermagem pode contribuir para que enfermeiros da prática clínica tenham a clareza de “quais intervenções de enfermagem fornecer e qual a quantidade de cada intervenção devem ser fornecidos para atingir o resultado desejado”^(5)^, minimizando a variabilidade da assistência prestada aos pacientes. Para isso, é igualmente importante incluir discussões que abordem as atividades de enfermagem integradas a essas intervenções, visto que as estruturam.

A segunda perspectiva, ou seja, a da gestão de pessoal de enfermagem, pode ser encontrada a partir do termo “dose de enfermagem”, definido como “o nível (ou seja, número e tipo) de equipe de enfermagem necessária para fornecer cuidados que produzem os resultados pretendidos para o paciente”^(6)^. Nesse contexto, para os autores, a “dose de enfermagem” seria “uma variável estrutural que captura a capacidade das enfermeiras de fornecer cuidados de alta qualidade em hospitais de cuidados agudos”^(6)^.

À luz dos autores acima citados, dois atributos refletem o conceito de dose de enfermagem: ingredientes ativos e intensidade. O primeiro representaria os elementos essenciais e diferenciadores dos enfermeiros em relação aos demais profissionais de saúde, e teria como indicadores críticos conhecimento teórico e prático dos enfermeiros, e combinação de habilidades. Já intensidade representaria o potencial para interações enfermeiro-paciente, sendo operacionalizada em termos de quantidade e frequência^(6)^. A perspectiva sob a qual serão realizadas as reflexões neste estudo se aproxima da compreensão do termo “dose da intervenção de enfermagem” na relação com a assistência de enfermagem e as suas repercussões^(5)^.

A literatura tem apontado com preocupação a questão da dose da intervenção de enfermagem, “ainda mais, se pensarmos no caminho a ser percorrido na estruturação da clínica da enfermagem, no desenvolvimento de pesquisas clínicas solidificadas, o monitoramento da dosagem da intervenção de enfermagem é crucial. Carece da demonstração da eficácia e efetividade das intervenções, das especificidades da dose terapêutica empregada”^(7)^ diante de um fenômeno identificado como passível de tratamento por enfermeiros.

Diante do exposto, espera-se que este estudo contribua para a prática profissional e auxilie para que a tomada de decisão terapêutica contemple todos os elementos prescritivos, quais sejam: data da elaboração da prescrição; verbo declarador da ação a ser realizada; quem deverá realizá-la: o sujeito; a frase descritiva que contemple como, quando, onde, com que frequência, por quanto tempo ou quanto; além da assinatura do prescritor^(8)^. Estudo de revisão apontou dificuldade em discernir a dose da intervenção nos estudos revisados para suportar a replicabilidade^(9)^. Discutir a partir da perspectiva “por quanto tempo ou quanto?”^(8)^ pode auxiliar a descortinar tomada de decisão terapêutica em enfermagem mais acurada e replicável. Assim, objetivou-se compreender as repercussões da dose da intervenção de enfermagem para a prática profissional.

## MÉTODO

Trata-se de estudo de reflexão teórica, elaborado a partir de um *corpus* de textos selecionados por conveniência que subsidiaram a compreensão da problemática aventada, ou seja, a compreensão das repercussões da dose da intervenção de enfermagem para a prática profissional. O exercício reflexivo iniciou-se a partir da seguinte questão: quais são as repercussões da dose da intervenção de enfermagem para a prática profissional? Considerou-se que, no cotidiano assistencial, as atividades de enfermagem estão no menor nível de abstração e maior concretude, e são parte integrante e indissociável das intervenções de enfermagem, conforme o sistema de linguagem padronizada adotado nesta reflexão, ou seja, a Classificação das Intervenções de Enfermagem^(4)^, partindo-se das atividades para compreender as repercussões da intervenção no que diz respeito à dose da intervenção de enfermagem. Os diagnósticos de enfermagem foram considerados a partir dos diagnósticos de enfermagem da NANDA-Internacional^(3)^.

Considerando-se as questões éticas, o estudo dispensa submissão à Comitê de Ética em Pesquisa, contudo assegurou-se o acesso às fontes de pesquisa de forma ética, garantindo a integridade científica a partir delas, bem como boas práticas para a produção de conhecimento apresentado nesta reflexão teórica. Para tanto, delineou-se a temática reflexiva “Repercussões da dose da intervenção de enfermagem para a prática profissional”.

### Repercussões da Dose da Intervenção de Enfermagem Para a Prática Profissional

Para melhor contextualizar a problemática aventada, lança-se mão de um caso clínico simulado, no contexto da enfermagem perioperatória, tendo como fenômeno de interesse a dor:

Idosa, 67 anos, submetida com urgência a uma cirurgia bem-sucedida para tratar uma fratura do colo femoral em 23 de agosto de 2024 por volta das 23:00. Em 24 de agosto, no primeiro horário da manhã, às 06:00, a paciente comunica ao enfermeiro plantonista que sente dor no local em que foi realizado o procedimento cirúrgico, no momento em que ele passa a ronda de vigília clínica de seus pacientes. Na ocasião, a paciente fora avaliada por meio da Escala Verbal Numérica (EVN), referindo o nível de sua dor em 8 (0–10). No seguimento, o enfermeiro revisou a prescrição médica e identificou nela o item 3 – dipirona 1,0 g – endovenosa (EV) 6/6 horas para às 07:00. Tomadas as medidas relacionadas à prática segura para administração de medicamentos, administrou-se o item prescrito, e a paciente, cerca de 35 minutos após ser medicada, já referira um nível de dor em 4 pontos na EVN, comparado à primeira avaliação. Após 60 minutos, refere um leve incômodo, que quantificara em 1 ponto na EVN, por ocasião da reavaliação pelo enfermeiro.

A partir do caso clínico simulado, cumpre destacar que a dipirona, a depender da idade do cliente, do peso ou mesmo da via de administração, terá dosagens diferentes e tempos de início de ação também diferenciados: para adultos e adolescentes acima de 15 anos (> 31 kg), consideram-se doses entre 500 e 750 mg, por via oral de 1 – 4 vezes/dia, e doses entre 1.000 e 2.500 mg, por via intramuscular ou endovenosa, de 1 – 4 vezes/dia. Em pediatria (acima de 3 meses), por exemplo, a dose por via oral em crianças de até 30 kg deve situar-se entre 10 e 15 mg/kg até 4 vezes/dia. A dipirona está indicada como tratamento de manifestações dolorosas e febre^(10)^.

Compreende-se, a partir do parágrafo anterior, que a intervenção colaborativa administração de analgésico^(4)^ está sustentada em critérios prescritivos claros (faixa etária, peso do cliente e via de administração) para o medicamento (tratamento) em questão, ou seja, doses mínimas e máximas, além do intervalo de administração entre as doses, que foram selecionados a partir de tais critérios. Partindo-se do caso clínico em questão, pondera-se que o enfermeiro deve buscar, semelhantemente, critérios posológicos claros para a assistência de enfermagem, de modo que esses critérios lhe permitam intervir autonomamente nos fenômenos de particular interesse à enfermagem e circunscrever, de forma objetiva, como prescrevem tratamentos a partir das intervenções de enfermagem e das dosagens que elas podem abarcar.

À luz do conhecimento disciplinar – base da prática autônoma do enfermeiro –, ele deve ser capaz de compreender o “o que” a enfermagem conhece – diagnósticos de enfermagem–, “como” trata esse “o que” – intervenções de enfermagem–, bem como os potenciais desfechos clínicos – resultados esperados e exequíveis de enfermagem e de saúde^(1)^. Para influenciar tais resultados, necessariamente, é preciso considerar “o quanto” se implementa do “como”, a partir de um determinado “o que”— dose da intervenção de enfermagem. Esta seria a base para melhorar resultados esperados e exequíveis^(1)^, além de modificar desfechos clínicos em enfermagem autonomamente, descortinando as relações entre os elementos da assistência de enfermagem no plano assistencial de enfermagem. Deve-se ponderar, também, que as intervenções de enfermagem se relacionam de modo a modificar comportamentos de saúde. Nesse sentido, cabe refletir: como dosar informações no contexto da educação em saúde?

Uma vez identificado um diagnóstico, estabelece-se um resultado esperado e exequível^(1)^ inicial, uma “foto inicial”^1^ da realidade a ser abordada. Tendo em mãos essa “foto inicial”, o enfermeiro lança mão de intervenções de enfermagem para tratar o diagnóstico de enfermagem prioritário ou “diagnóstico de trabalho”^2^, de modo a impactar os potenciais desfechos clínicos. A partir das reavaliações, terá “fotos parciais” e/ou “fotos finais” – respostas dos clientes aos tratamentos prescritos e implementados. O “diagnóstico de trabalho”, nesta reflexão, pode ser compreendido como o fenômeno de enfermagem a ser abordado em um dado recorte temporal no plano assistencial de enfermagem; por exemplo, ao longo de 12 ou 24 horas de um plantão, apesar de outras possibilidades diagnósticas.

É a partir do “diagnóstico de trabalho” que o enfermeiro irá analisar e estabelecer como o paciente se comportou diante do plano assistencial de enfermagem planejado e implementado, permitindo que os desfechos clínicos de curto, médio e longo prazos sejam identificados e acompanhados – base para a evolução de enfermagem. É importante que se tenha em mente que os resultados esperados e exequíveis^(1)^ iniciais advêm da resposta humana identificada, e a depender de seu tipo, deverão ser melhorados, diminuídos, mantidos ou outra classificação que lhes couber. À vista disso, resultados iniciais, a partir de um corte temporal avaliativo, ganharão a conotação de parciais ou finais quando reavaliados, ação característica da evolução de enfermagem.

A identificação de “problemas existentes, condições de vulnerabilidades ou disposições para melhorar comportamentos de saúde”^(1)^ será a base para a tomada de decisão terapêutica em enfermagem. Problemas existentes e/ou condições de vulnerabilidade podem expressar diferentes graus de gravidade, como extensão de apresentação do fenômeno identificado, desafiando enfermeiros a selecionar a dose da intervenção de enfermagem conforme a gravidade expressa por cada cliente, pois o tratamento e a dose selecionados e prescritos, tendo como referente o fenômeno identificado, devem ter a capacidade de reverter, estabilizar e/ou manter uma determinada condição clínica priorizada – um diagnóstico de enfermagem. A perspectiva de um *status* para o diagnóstico de enfermagem ancora-se na literatura de enfermagem^(7,11)^.

Também, a partir da perspectiva de gestão de riscos assistenciais, as intervenções de enfermagem e, como consequência, as doses selecionadas e prescritas, deverão ser capazes de realizar a adequada gestão de riscos clínicos identificados. Suponha-se que um dado paciente é internado em uma unidade hospitalar e, com base em um instrumento de avaliação de risco^(12)^, um enfermeiro identifica que ele tenha um alto risco para quedas, pois alcançou o escore de 14 pontos na avaliação, então, no momento seguinte, é internado um outro paciente, que pontuou 10 pontos, e foi classificado como tendo um moderado risco para quedas, o enfermeiro responsável pelos dois planos assistenciais de enfermagem seleciona a intervenção prevenção contra quedas^(4)^, definida como “instituição de precauções especiais em paciente em risco de ferimentos devido à queda”^(4)^. Compreende-se, a partir dos escores alcançados, que embora representem rótulos diagnósticos semelhantes – risco de quedas no adulto^(3)^–, a gravidade do fenômeno apresentou-se diferente, justificada por diferentes escores e, portanto, requererão doses da intervenção de enfermagem considerando “posologias” diferentes.

Ampliando as reflexões a partir das situações clínicas simuladas anteriores, no primeiro caso, trata-se de um paciente de 70 anos com nível superior de escolaridade, contador aposentado, mas pouco ativo e dependente para o autocuidado. Ademais, ele demonstrou certa resistência às orientações e nega o risco. No segundo exemplo, trata-se de outro idoso, agora com 75 anos, ativo, que, apesar de menor escolaridade, demonstra maior adesão às orientações preventivas. Ambos os pacientes possuem o diagnóstico de risco de quedas no adulto^(3)^, porém expressam-no clinicamente de modo diverso. A partir dessa provocação, questiona-se: qual a dose da intervenção de enfermagem que deverá ser prescrita em forma de orientação e vigilância clínica para ambos os pacientes, visto que o risco se apresenta em intensidades ou graus diferentes? Embora o contexto socioambiental adotado nos exemplos acima seja o hospitalar, é possível extrapolar as reflexões deste texto para outros cenários, tais como a Atenção Primária à Saúde, visto que é o fenômeno priorizado, bem como suas características clínicas, que serão mandatórias para a seleção da dose da intervenção de enfermagem.

Inspirando-se em literatura pertinente^(1,3–7,11)^, compreende-se que a dose de intervenção de enfermagem diz respeito à quantidade ou intensidade necessária para que uma dada intervenção de enfermagem, quando implementada, alcance o efeito esperado, de modo a tratar uma pessoa, família, coletividade ou grupos especiais que apresentam uma determinada resposta humana, influenciando resultados esperados e exequíveis de enfermagem e de saúde, e modificando ou mantendo desfechos clínicos de enfermagem pela alteração do *status* – presente, melhorado, piorado, inalterado ou resolvido – de um dado diagnóstico de enfermagem prioritário ou de trabalho em um dado contexto socioambiental. Intervenções que contemplem aspectos quantificáveis, representados por mililitros (ml), miligramas (mg), centímetros (cm), entre outras medidas, deverão ter sua dose considerada a partir desse tipo de parâmetro, expressando de modo objetivo a perspectiva de dose da intervenção a ser prescrita e implementada.

Quando intervenções de enfermagem envolverem aspectos comportamentais e/ou atitudinais, tendo como base prescritiva a interação entre o enfermeiro e o cliente, infere-se que a dose da intervenção de enfermagem seja compreendida a partir de medidas de intensidade e regularidade interativas, pois guardam estreita relação com o fenômeno da repetição e/ou reforço da oferta da intervenção. Por exemplo, aquelas intervenções de enfermagem que contemplem orientações de educação em saúde, visto que a dose da intervenção tem a ver com modificações ou manutenção de comportamentos de saúde expressos pela pessoa, família, coletividade ou grupos especiais que podem demandar o reforço dessas mesmas orientações.

A abordagem teórico-prática do fenômeno dose da intervenção de enfermagem, nessa perspectiva, reveste-se de um complexo movimento norteado pela interação enfermeiro-pessoa, família, coletividade ou grupos especiais, e encerra em seu bojo um componente relacional próprio que, para ser compreendido, demanda um encontro frente a frente autêntico, em que ambos os envolvidos tenham voz e espaço de fala contributiva. Compreende-se o desafio de se estabelecer a dose da intervenção de enfermagem à luz do fenômeno interativo de uma orientação em saúde, pois o contexto e a cultura de cada pessoa são óticas sensíveis de interpretação do mundo e das interações que nele se processam. Portanto, são fatores que devem ser considerados na seleção, prescrição e implementação de ações de enfermagem, ou seja, na tomada de decisão terapêutica em enfermagem, que deve considerar também a dose da intervenção de enfermagem.

Descortina-se, nesse sentido, uma práxis de enfermagem relacional e humana única no contexto das ciências da saúde, sem a qual não se pode conhecer a dose ou mesmo se estabelecer a posologia dela, a qual é permeada, entre outros aspectos, pelo respeito mútuo e reconhecimento dos seres em interação, em um dado contexto socioambiental norteado pela prática educativa. A intensidade de cada intervenção será avaliada *in loco* e no momento em que o enfermeiro estiver pondo-a em prática à beira do leito ou em outro cenário qualquer, pois o *feedback*, o nível de compreensão de uma dada orientação ou mesmo a capacidade de reprodução a partir do que foi orientado serão mandatórios para se compreender que dose da intervenção de enfermagem nos diversos contextos educativos interacionais deve ser considerada.

Semelhantemente a qualquer outro tratamento, deve-se considerar que as intervenções de enfermagem, a partir de sua implementação, têm a possibilidade do surgimento de efeitos colaterais que nem sempre estarão perceptíveis “a olho nu”, pois intervenções de enfermagem compreendem uma carga de interação importante. Assim, há que discutir e estressar este tema até as últimas consequências, para que tanto a dose quanto os resultantes de sua implementação sejam considerados em uma totalidade, na qual os efeitos colaterais e/ou eventos adversos sejam considerados. Uma possibilidade de se compreender como repercutem intervenções de enfermagem no contexto interacional seria as sensações de bem-estar ou não resultantes das ações postas em prática, a partir de prescrições de enfermagem. Nesse sentido, a comunicação verbal e não verbal é imprescindível de ser considerada.

A delimitação de campo de atuação inequívoco como de enfermagem, que caracterize de modo uníssono o que se defende como “cuidado de enfermagem”, pode sucumbir no excesso de prescrições que valorizem ações de vigilância e rotina assistencial diária, compreendidas por vezes como cuidados de enfermagem embebidos, em muitos cenários assistenciais, de aspectos procedimentais tão somente. A vigilância clínica é parte da assistência de enfermagem, mas ações que considerem o tratamento clínico modificador de fato são mandatórias para que se delimite cada vez mais o campo de atuação profissional disciplinar. A vigilância clínica é um componente atitudinal das intervenções de enfermagem, mas não deve ser a base exclusiva de tratamentos de enfermagem.

Há que avançar para além de verbos como “monitorar”, “acompanhar” e “observar”, que guardam em si uma conotação de vigília clínica estrita, para verbos que assumam o caráter prescritivo/resolutivo, tais como “realizar”, “administrar”, “orientar”, “implementar”, “desbridar”, “retirar” e “acrescentar”, que se aproximam de cursos de ação e saber-fazer autônomos, transpondo ações de encaminhamento. Nesse sentido, teorias de enfermagem podem auxiliar na delimitação do caráter particular de fenômenos clínicos, como os de enfermagem; por isso, além de auxiliar a declarar o que é singular à enfermagem, compreendem, nessa mesma delimitação, como tratar pela particularização de como recortam tais fenômenos como os de enfermagem.

A seleção da dose da intervenção de enfermagem tem relação com a *expertise* clínica individual, estando relacionada à capacidade de compreender a gravidade do diagnóstico de enfermagem identificado, o contexto socioambiental em que ele ocorre e os potenciais desfechos clínicos esperados a curto, médio e longo prazo, mas também à proposta terapêutica à luz do plano assistencial multiprofissional, pois, a depender do perfil do paciente, seja um paciente clínico ou cirúrgico, em cuidados de reabilitação, em cuidados crônicos ou mesmo em cuidados paliativos, irão influenciar a posologia da dose da intervenção de enfermagem. A dose irá variar conforme a extensão da apresentação dos fenômenos clínicos identificados.

Nesta reflexão, o conceito de intervenção está suportado na Classificação das Intervenções de Enfermagem, ou seja, “qualquer **tratamento** (grifo nosso) que, baseado em julgamento e conhecimento clínico, um enfermeiro ponha em prática para melhorar os resultados do paciente”^(4)^. Os resultados esperados e exequíveis^(1)^ serão melhorados à medida que o tratamento prescrito e implementado modifique o *status* do diagnóstico de enfermagem “de trabalho”, expresso a partir de modificações ou manutenção de sinais e sintomas de importância clínica para a enfermagem. Nesse sentido, é imprescindível compreender que diagnósticos de enfermagem podem estar presentes, melhorados, piorados, inalterados e resolvidos^(11)^.

Na evolução de enfermagem, 5ª etapa do PE, perguntas do tipo “como o paciente respondeu ao tratamento nas últimas 12 ou 24 horas?” são orientadoras para processos avaliativos acurados que permitam corrigir rotas no plano assistencial de enfermagem, diante de um prognóstico clínico favorável ou desfavorável identificado, ou seja, a “predição do curso de uma doença e é expresso como a probabilidade de um evento particular vir a ocorrer no futuro. As predições são baseadas em grupos definidos de pacientes e o desfecho pode ser diferente para cada indivíduo. Entretanto, o conhecimento dos prováveis prognósticos é útil na determinação do melhor tratamento. Fatores prognósticos são características associadas com o desfecho em pacientes com a doença em questão”^(13)^; no caso da enfermagem, como se comportam as respostas humanas em um dado contexto e tempo para se avaliar e definir o prognóstico.

Compreende-se que, no plano assistencial de enfermagem, não basta selecionar, prescrever e implementar intervenções de enfermagem, há que se discutir o “quanto” de cada intervenção deverá ser “administrado” e em que período. Não raras vezes, nas prescrições de enfermagem em muitas instituições de saúde, destacando-se as hospitalares, a prescrição de enfermagem é operacionalizada por dois itens principais: uma frase no infinitivo, que determina a atividade a ser realizada (o que será feito); e um horário (periodicidade ou intervalo de realização de uma dada ação). Contudo, enfermeiros e suas equipes devem orientar suas ações a partir de perguntas como “quanto de cuidado de enfermagem deverá ser dispensado e em qual intensidade?”.

Por atividades de enfermagem, compreende-se que estejam “no nível concreto de ação, pois são as condutas ou ações específicas tomadas para implementar uma intervenção e que auxiliam os pacientes a progredirem em direção ao resultado esperado”^(4)^. As atividades que compõem cada intervenção podem ser consideradas passos que, postos em prática, fornecem um caminho para implementar uma dada intervenção. Embora a literatura aponte que as pesquisas poderiam lançar mão das atividades como preditoras da dose da intervenção, compreende-se que contabilizar um dado número de atividades como base para definir uma dada dose não contempla a compreensão de dose de enfermagem discutida nesta reflexão.

O Quadro 1 apresenta a intervenção de enfermagem “Prevenção de lesão por pressão”^(4)^ e as atividades que a estruturam - base para o exercício reflexivo acerca da dose da intervenção de enfermagem que se quer propor neste artigo. Em cada atividade, buscou-se a compreensão das potenciais doses ou não a serem consideradas. Em algumas atividades, pela forma como estão descritas, contemplam mais de uma atividade em seu texto, o que dificultaria a seleção precisa de uma dose. Compreende-se também que a Classificação das Intervenções de Enfermagem não tem este tipo de compromisso – um excessivo nível de descrição que, no entender dos autores desta reflexão, não seria a proposta da classificação. Contudo, o exercício reflexivo realizado auxilia a descortinar a problemática que se quer discutir.

**Quadro 1:** Reflexões teóricas a partir de uma intervenção de enfermagem – Niterói, RJ, Brasil, 2024.

Intervenção - Prevenção de lesões por pressão **Definição: prevenção de lesões por pressão para um indivíduo com alto risco de desenvolvê-las.**	
**Atividades**	**Exercício crítico-reflexivo**
1. Utilizar uma ferramenta de avaliação de risco que seja reconhecida para monitorar os fatores de risco do indivíduo (p. ex., escala de Braden).	1. Ferramentas de avaliação de riscos auxiliam a compreender a gravidade deles e a definir a intensidade da prevenção a ser prescrita/implementada. Neste caso, a dose estaria relacionada ao intervalo de avaliação e à vigilância clínica implementada.
2. Utilizar métodos de medir a temperatura da pele para determinar o risco de lesões por pressão, de acordo com o protocolo da instituição.	2. Métodos de mensuração auxiliam a compreender a gravidade do risco e a definir a intensidade da prevenção a ser prescrita. Nesse sentido, aqui caberia o intervalo de avaliação, que suportaria a seleção da dose ou dos recursos preventivos a serem selecionados.
3. Encorajar o indivíduo a não fumar e a evitar o uso de álcool.	3. Esta atividade diz respeito às ações de orientação para a cessação do tabagismo e uso de álcool. Envolvem a adesão às medidas orientadoras e precisam considerar o nível de conhecimento e engajamento para a cessação do tabagismo. A dose aqui está relacionada à quantidade/intensidade de informação a ser partilhada, bem como ao número de abordagens consideradas. A compreensão e a modificação do comportamento a partir da primeira consulta de enfermagem seriam a base da seleção da dose, reavaliada em outros encontros, pois há que se considerar o quanto o indivíduo estará disposto a modificar os comportamentos de risco.
4. Documentar quaisquer incidências prévias de formação de lesões por pressão.	4. A documentação faz parte e é resultante da avaliação inicial e contínua, e possibilita a compreensão de como se deu o tratamento de enfermagem em momento anterior. Não cabe aqui considerar dose da intervenção de enfermagem, mas a qualidade da avaliação inicial e dos registros profissionais dela resultantes.
5. Documentar o peso e as mudanças de peso.	5. Mudanças no peso podem orientar a intensidade de medidas preventivas e, como consequência, a dose da intervenção de enfermagem, pois, a depender do grau de risco, uma série de tecnologias e ações de enfermagem deverão ser consideradas.
6. Documentar o estado da pele na admissão e diariamente.	6. A avaliação inicial da pele e a resposta dela ao tratamento prescrito podem auxiliar no escalonamento e seleção de medidas preventivas e tratamentos. Desse modo, existe uma correlação direta entre o estado atual da pele, seu risco clínico potencial para acometimento de uma dada lesão e os desfechos clínicos esperados. Contudo, a atividade diz respeito ao registro profissional.
7. Monitorar intensivamente áreas avermelhadas.	7. O monitoramento tem relação com a vigília clínica de enfermagem e deve ser compreendido, nessa realidade, como dose, tendo relação com a intensidade com que o monitoramento é feito, guardando relação direta com o intervalo de tempo e o tempo dispendido para a avaliação.
8. Remover a umidade excessiva da pele que resulta de perspiração, drenagem de feridas, e incontinência fecal e urinária.	8. Observam-se aqui diversos fatores que contribuem para a umidade excessiva, o que dificulta estabelecer uma dose considerando a atividade de enfermagem em questão, pois a exposição à umidade pode ocorrer em momentos diferentes e a partir de diferentes causas. Caberia discutir na prescrição a dose relacionada a um monitoramento contínuo ou à individualização de cada problema/ação/dose.
9. Aplicar barreiras protetoras, como cremes ou materiais absorventes de umidade, para remover o excesso de umidade, conforme apropriado.	9. Para além da remoção da umidade, há que se questionar quanto de creme barreira deve ser aplicado, pois isso se relaciona à dose da intervenção de enfermagem. Ademais, a atividade também cita materiais absorventes que teriam uma outra perspectiva de dose, como ligada à sua capacidade de absorção e, como consequência, horas de proteção que a tecnologia em questão pode ofertar pela capacidade de retenção de um dado volume de urina.
10. Modificar o posicionamento a cada 1 a 2 horas.	10. O reposicionamento é uma das atividades que qualifica de forma sensível a compreensão do que seja dose da intervenção de enfermagem, pois identifica-se que a dose está ligada ao intervalo em que ela ocorre, ou seja, a cada 1 a 2 horas, será dispensada uma “dose de mudança de decúbito” para prevenir o aparecimento de lesões por pressão que, entre outros fatores, estão relacionadas a quanto um dado tecido pode estar vivenciando de isquemia local.
11. Realizar a mudança na posição do paciente com cuidado (evitar deformação por forças tangenciais) para prevenir lesão da pele frágil.	11. Esta atividade indica a forma de fazer e tem relação com a atividade “Modificar o posicionamento a cada 1 a 2 horas”; portanto, não caberia discutir dose da intervenção de enfermagem neste item, mas a qualidade com que uma dada ação de cuidado é realizada.
12. Colocar o roteiro de mudanças de posição ao lado do leito, conforme apropriado.	12. A indicação do roteiro serve como base para que o paciente não repita a posição antes do tempo indicado; portanto, não cabe discutir a dose da intervenção de enfermagem neste item, já que a estratégia serviria aos profissionais como guia para a atividade.
13. Inspecionar a pele sobre proeminências ósseas e outros pontos de pressão pelo menos 1 vez/dia durante os reposicionamentos.	13. O monitoramento tem a ver com a intensidade diante de um risco potencial identificado em um paciente; por isso, a depender do nível de risco, a dose de monitoramento deverá ser reajustada a partir da avaliação da pele a cada reavaliação, ou seja, o quanto de monitoramento deverá ser prescrito. No caso desta atividade, a relação entre ação e dose tem a ver com o intervalo de tempo em que as ações são realizadas.
14. Evitar massagear sobre proeminências ósseas.	14. Orientação de boa prática clínica que complementa a forma de intervir. Não cabe discutir a dose da intervenção de enfermagem.
15. Posicionar com travesseiros para elevar pontos de pressão do leito.	15. Orientação de boa prática clínica que complementa a forma de intervir. Não cabe discutir a dose da intervenção de enfermagem.
16. Manter os lençóis limpos, secos e sem amassados.	16. Orientação de boa prática clínica que complementa a forma de intervir. Não cabe discutir a dose da intervenção de enfermagem.
17. Fazer a cama com pregas para dedos dos pés.	17. Orientação de boa prática clínica que complementa a forma de intervir. Não cabe discutir a dose da intervenção de enfermagem.
18. Utilizar leitos e colchões especializados, conforme apropriado.	18. A utilização de leitos e superfícies especializados deverá ser selecionada com base no grau ou escore de risco identificado a partir de um dado instrumento de avaliação clínica e, nesse sentido, tais leitos e colchões especializados serão selecionados conforme tal gravidade. Isto se relaciona à “quantidade” de tecnologias disponíveis.
19. Utilizar equipamentos no leito que protejam o paciente.	19. Orientação de boa prática clínica que complementa a forma de intervir. Não cabe discutir a dose da intervenção de enfermagem.
20. Evitar equipamentos tipo “rosquinha” na área sacral.	20. Orientação de boa prática clínica que complementa a forma de intervir. Não cabe discutir a dose da intervenção de enfermagem.
21. Hidratar a pele ressecada, ainda íntegra.	21. A hidratação tem relação com o aparecimento de lesões por pressão; portanto, cabe discutir a dose da hidratação a ser prescrita. As características do produto selecionado serão mandatórias para a seleção da dose da intervenção de enfermagem.
22. Evitar água quente e utilizar sabão neutro durante os banhos.	22. Orientação de boa prática clínica que complementa a forma de intervir. Não cabe discutir a dose da intervenção de enfermagem.
23. Monitorar para fontes de pressão e atrito.	23. A dose de monitoramento terá relação com a gravidade do risco de acometimento por lesões por pressão. Um intervalo entre os monitoramentos pode estar relacionado à dose no sentido de quanto acompanhamento será implementado.
24. Aplicar protetores nos calcanhares e cotovelos, conforme apropriado.	24. A aplicação de protetores tem a ver com o grau de risco, pois, a depender do tipo de risco, seleciona-se uma dada tecnologia que dê conta de proteger o paciente avaliado e a sua pele. Assim, seguir as orientações do fabricante é importante. Tratando-se de cremes e *sprays*, a quantidade dispensada na aplicação deverá ser considerada como a dose da intervenção de enfermagem. Importante destacar que a seleção da quantidade de creme barreira ou protetor tem a ver com a extensão da superfície em risco.
25. Facilitar pequenas trocas frequentes na posição do paciente.	25. Os pequenos reposicionamentos serão considerados diante do risco de lesão por pressão identificado; portanto, a dose tem relação com os intervalos de troca e o grau de risco. Neste entendimento, a dose da intervenção de enfermagem e o intervalo de troca de posição são indissociáveis.
26. Providenciar um trapézio para auxiliar nas mudanças frequentes da posição do paciente.	26. Orientação de boa prática clínica que complementa a forma de intervir. Não cabe discutir a dose da intervenção de enfermagem.
27. Monitorar a mobilidade e a atividade do indivíduo.	27. Os níveis de mobilidade e atividade serão mandatórios para a intensidade de implementação de medidas preventivas e, portanto, norteadores na definição da dose da intervenção de enfermagem no que diz respeito ao reposicionamento do paciente.
28. Assegurar ingestão adequada da dieta, especialmente de proteínas, vitaminas B e C, ferro e calorias, utilizando suplementos, conforme apropriado.	28. Atividade colaborativa que, para ser implementada, deverá ser discutida junto a nutrólogos e nutricionistas. Nesse sentido, a dose da intervenção deverá ser discutida conjuntamente, mas acompanhada de perto pelo enfermeiro e pela equipe de enfermagem, pois são os profissionais que comumente administram ou ofertam alimentos aos pacientes.
29. Auxiliar o indivíduo a manter um peso saudável.	29. A manutenção do peso saudável é uma atividade complexa que abarca uma série de atividades, e o monitoramento do peso é uma medida possível de ser implementada de forma rápida e independente, mas não deve ser a única. O enfermeiro precisa trabalhar em conjunto por meio de um plano terapêutico multiprofissional para auxiliar o indivíduo na manutenção do peso adequado para as variáveis definidas em evidências científicas. A dose desta atividade está relacionada à quantidade de orientação que seria dispensada para que um indivíduo perca peso.
30. Instruir a família ou cuidador sobre sinais de lesão da pele, conforme apropriado.	30. Esta atividade está relacionada à aprendizagem e engajamento de comportamentos de prevenção de lesão por pressão estabelecidos por todos os envolvidos. A dosagem da intervenção está ligada à quantidade de informação partilhada, à intensidade da abordagem e ao intervalo entre as orientações, ou seja, o intervalo entre as doses de informação ofertada.

Fonte: Adaptado de Classificação das Intervenções de Enfermagem^(4)^.

O exercício reflexivo anterior descortinou a complexa, mas necessária, discussão da dose da intervenção de enfermagem no nível das atividades ou ações concretas, visto que serão elas prescritas e implementadas nos cenários assistenciais pelo enfermeiro e equipe, e que guiarão as ações assistenciais que elas representam por meio da prescrição de enfermagem. Neste sentido, apontar cursos de ação é indiscutível, como também o é descrever claramente em que medida essas mesmas ações deverão ser realizadas – o quanto do como. Tratamentos necessariamente devem considerar doses a serem prescritas.

No contexto do cuidado de enfermagem, famílias e pessoas significativas são importantes instrumentos terapêuticos de que os pacientes dispõem para dar continuidade a tratamentos ou mesmo prevenir complicações de saúde, desdobrando-se em parceiros - peças fundamentais que deverão ser incorporadas no momento da seleção e implementação de ações em saúde e enfermagem. Assim, avaliar de forma crítica como familiares e pessoas significativas compreendem o plano assistencial de enfermagem é imprescindível, pois, na seleção de intervenções de enfermagem e suas respectivas doses, eles deverão ser considerados.

A limitação desta pesquisa está no contexto de sua abordagem, ou seja, uma reflexão teórica, pois a complexidade da problemática aqui discutida poderia ser considerada por métodos mais robustos. A pesquisa respondeu ao objetivo quando, por meio do exercício reflexivo proposto, identificou as ­potenciais repercussões da dose da intervenção de enfermagem para a ­prática profissional.

### Considerações Finais

Para compreender a dose da intervenção de enfermagem e sua repercussão na prática profissional, é necessário considerar que sua prescrição deve ser baseada em critérios posológicos claros. Sem esses critérios, torna-se pouco provável, no cotidiano assistencial, determinar a dosagem da intervenção e/ou medir os impactos dos tratamentos prescritos por enfermeiros nos desfechos clínicos dos pacientes. A perspectiva da dose da intervenção de enfermagem apresentada neste texto está delimitada pela Classificação das Intervenções de Enfermagem. Em essência, um conjunto de atividades compõe uma intervenção de enfermagem. Compreender como cada atividade pode requerer uma dose específica, e como o conjunto integrado de atividades expressa a dose de uma intervenção como um todo, é fundamental para compreender suas repercussões na prática profissional em enfermagem.

À luz do exercício reflexivo realizado e do *corpus* de texto selecionado, as repercussões delineadas incluem: a possibilidade de mensurar a eficácia e efetividade dos tratamentos prescritos por enfermeiros; a redução da variabilidade nas ações prescritas, pois será possível não apenas escolher um curso de ação, mas também determiná-lo, levando-se em conta parâmetros como frequência, intensidade e quantidade; maior clareza para auxiliares e técnicos de enfermagem sobre os padrões de prática estabelecidos pelos enfermeiros; a construção de uma base mais sólida de evidências para que enfermeiros, em diversos contextos, sintam-se seguros ao assumir a responsabilidade por desfechos clínicos em enfermagem; maior replicabilidade das intervenções/atividades em estudos de intervenção de enfermagem.

Ademais, incluem-se maior clareza sobre o delineamento das ações autônomas do enfermeiro como parte da equipe de saúde e aumento do potencial para replicabilidade de tratamentos prescritos em situações semelhantes, o que pode contribuir para a aquisição de *expertise* clínica em processos de tomada de decisão diagnóstica ou terapêutica. Por fim, há maior clareza para futuras atualizações da Classificação das Intervenções de Enfermagem e melhores orientações sobre como prescrever a assistência de maneira eficaz, impactando os resultados esperados e exequíveis e os *status* dos diagnósticos priorizados no contexto da prática clínica.

## Data Availability

Todo o conjunto de dados que dá suporte aos resultados deste estudo foi publicado no próprio artigo.

## Footnotes

1 A associação de resultados esperados de enfermagem a registros fotográficos ou fotos “iniciais”, “parciais” e “finais” remete à ação de fotografar feridas ou lesões, para que se tenha um parâmetro inicial e, a partir dele, se possa estabelecer a evolução delas em um dado contexto assistencial, conforme um período estabelecido, permitindo avaliar os resultados esperados e os alcançados.

2 A expressão “diagnóstico de trabalho” foi ouvida pela primeira vez pelo primeiro autor desta reflexão, quando ele conversava com a Professora Doutora Maria Márcia Bachion em um evento em comemoração ao aniversário do Grupo de Estudos em Sistematização da Assistência de Enfermagem, da Universidade Federal Fluminense (GESAE-UFF), ocorrido no Rio de Janeiro.

# Translation: Dosis de intervención de enfermería: repercusiones en lapráctica professional

**DOI:** 10.1590/1980-220X-REEUSP-2025-0038es

**Authors:** George Luiz Alves Santos, Thiago Augusto Soares Monteiro da Silva

**Affiliations:** 1 Centro Universitário La Salle, Niterói, RJ, Brasil.; 2 Universidade Federal do Rio de Janeiro, Escola de Enfermagem Anna Nery, Rio de Janeiro, RJ, Brasil.; 3 Universidade de Vassouras, Vassouras, RJ, Brasil.

**Keywords:** Proceso de Enfermería, Razonamiento Clínico, Planificación de Atención al Paciente, Dosificación

## Abstract

El objetivo de este estudio fue comprender las repercusiones de la dosis de intervención enfermera en la práctica profesional. Se trata de un estudio de reflexión teórica que buscó responder a la siguiente pregunta: ¿cuáles son las repercusiones de la dosis de intervención enfermera en la práctica profesional? Para responder a esta pregunta, se consideró un corpus de textos seleccionados que permitieran comprender estas repercusiones. Se identificaron las siguientes repercusiones: la posibilidad de medir la eficacia y efectividad de los tratamientos prescritos por las enfermeras; la reducción de la variabilidad en las acciones prescritas; la construcción de una base de evidencia más sólida para que las enfermeras, en diferentes contextos, se sientan seguras de asumir la responsabilidad por los resultados clínicos en enfermería; la mayor replicabilidad de las acciones en estudios de intervención de enfermería; el mayor potencial de replicabilidad de los tratamientos prescritos en situaciones similares, entre otros. El objetivo del estudio se logró al identificar cómo la dosis de intervención de enfermería afecta la práctica profesional, sin la cual es imposible analizar el impacto de la atención en los resultados clínicos de los pacientes.

## INTRODUCCIÓN

Planificar la asistencia requiere que los enfermeros tengan en cuenta, esencialmente, tres elementos de la asistencia de enfermería, a saber, los diagnósticos, los resultados y las intervenciones de enfermería. Estos elementos pueden identificarse en la resolución actual, que trata de la implementación del Proceso de Enfermería (PE)^(1)^. La planificación de la enfermería se refiere al «desarrollo de un plan de asistencia dirigido a la persona, la familia, la colectividad, los grupos especiales, y compartido con los sujetos de los cuidados y el equipo de enfermería y salud»^(1)^. Para planificar a la luz de los elementos de la asistencia de enfermería, se deben priorizar determinados diagnósticos de enfermería, que serán obligatorios para la selección del tratamiento clínico más adecuado, debiéndose definir, siempre que sea posible, en colaboración con el cliente, la propuesta terapéutica: cura, rehabilitación, confort, prevención, promoción o incluso la reconfiguración de los cuidados cotidianos, en caso de dependencia parcial o total, así como los resultados previstos. En este sentido, la selección de los elementos más adecuados de la asistencia de enfermería presupone considerar tanto la toma de decisiones diagnósticas como terapéuticas, una perspectiva crítica también adecuada cuando se considera la evaluación de los resultados esperados y alcanzables en materia de enfermería y salud^(1)^.

En el contexto del equipo de salud, cada integrante necesita dilucidar los aspectos contributivos que delimitan su saber hacer disciplinario, mejor comprendidos cuando se piensa en un plan terapéutico multidisciplinar, del cual forma parte el plan de asistencia de enfermería. En este sentido, la contribución de cada miembro se demostrará a partir de cursos de acción especializados, decididos y acordados, siempre que sea posible, con el participante en la atención, idealmente en un ejercicio de planificación conjunta, poniendo de manifiesto la toma de decisiones terapéuticas individuales y colectivas. «Las decisiones terapéuticas, en el proceso de resolución de problemas clínicos, implican la idealización de planes o cursos de acción que tengan como objetivo cambiar la situación actual del problema por otra mejor»^(2)^, siempre que sea posible.

«Cada profesión en el ámbito de la salud tiene una manera de describir «lo que» sabe»^(3)^ y, en el caso particular de la enfermería, delimitada por su epistemología, lo que sabe se refiere a los diagnósticos de enfermería. Estos, por sus características, y a la luz del sistema de lenguaje estandarizado considerado en esta reflexión teórica, se clasifican en diagnósticos de enfermería centrados en el problema, en el riesgo o en la promoción de la salud^(3)^. En este sentido, se defiende que los enfermeros tratan «una respuesta humana a problemas de salud y/o procesos de la vida, o una susceptibilidad a tal respuesta»^(3)^. Cabe destacar, además, que sus conocimientos deben estar orientados por referencias teóricas propias de la enfermería, fundamentalmente para que las prácticas de salud y enfermería sean reconocidas, de manera inequívoca, como propias de los enfermeros, delineando la contribución clínico-asistencial de la enfermería en los diversos contextos socioambientales; solo así podrá consolidarse la episteme de la enfermería.

Más allá de las discusiones sobre «qué» se sabe—diagnóstico de enfermería—o incluso sobre «cómo» se actúa en relación con lo que se sabe^(3)^—intervención de enfermería—, como disciplina científica de las ciencias de la salud, sus representantes deben aclarar específicamente en qué medida actúan en relación con ese «cómo». Deben informar en sus planes asistenciales de enfermería la dosis de intervención de enfermería necesaria para tratar una determinada respuesta humana identificada como necesitada de intervención profesional, ponderando los tres tipos de presentaciones posibles considerados en este texto, a la luz del sistema de lenguaje estandarizado en particular, tales como diagnósticos de enfermería centrados en el problema, en el riesgo o en la promoción de la salud^(3)^.

Esta reflexión teórica se ocupa de comprender aspectos relacionados con el grado de actuación de los enfermeros a partir de la selección de un determinado «cómo» (la intervención de enfermería), pero no se puede ignorar el contexto en el que se inscribe dicha intervención (la implementación del PE), que comprende, además de las intervenciones, otras etapas, por lo que las consideraciones a lo largo del texto lo han tenido en cuenta. Para dilucidar «cómo» actúan, es necesario definir qué es una intervención de enfermería, ya que en la etapa de planificación a la que se refiere la actual resolución sobre el PE, se habla de «toma de decisión terapéutica»^(1)^, lo que supone adoptar un determinado curso de acción. Las intervenciones de enfermería se entienden como «cualquier tratamiento que, basado en el juicio y el conocimiento clínico, un enfermero pone en práctica para mejorar los resultados del paciente. Las intervenciones de enfermería incluyen tanto los cuidados directos como los indirectos»^(4)^.

La pertinencia de este tema radica en la escasez de estudios que aborden en profundidad la dosis de la intervención de enfermería, ya que «es importante determinar la dosis de una intervención para establecer su efecto en la práctica»^(4)^, sin embargo, «en la actualidad, no existe una solución al problema de la dosis de la intervención»^(4)^. Se han señalado como posibles soluciones a este impasse teórico-práctico el recuento del tiempo necesario para realizar una intervención, el recuento del número y la extensión de las actividades seleccionadas e implementadas en una intervención^(4)^.

En la literatura se identifican dos perspectivas teóricas para comprender la problemática de la dosificación: la asistencial y la de la gestión del personal de enfermería. Desde la primera perspectiva, se identifica el término «dosis de la intervención de enfermería», cuyos atributos esenciales serían la cantidad, la frecuencia y la duración de una intervención^(5)^. Sobre la implicación práctica del tema, los autores afirman que «medir y reportar la dosis de la intervención de enfermería en la investigación es esencial para el desarrollo de una base de evidencia adecuada para apoyar la práctica»^(5)^. A partir de Sidani y Braden (1998), los autores^(5)^ definen la cantidad como «la cantidad de intervención de enfermería prestada en un momento determinado»; la frecuencia como «el número de veces que se presta la intervención durante un período de tiempo especificado»; y la duración como «el período de tiempo completo durante el cual se presta la intervención».

La perspectiva adoptada por los autores para expresar la cantidad de intervenciones prestadas en el estudio anterior^(5)^ se basó en cada etiqueta de intervención de la Clasificación de Intervenciones de Enfermería, y no en las actividades que las componían—unidades de intervención—, definidas como «un conjunto de actividades realizadas al mismo tiempo y relacionadas con una única intervención»^(5)^. El enfoque dado por los autores a partir de la etiqueta de la intervención es estimulante, pero se entiende que cada etiqueta refleja la serie de actividades que la componen. En vista de ello, reflexionar sobre las actividades es considerar su papel determinante en la selección de la dosis, ya que influyen en la propia intervención de enfermería, al permitir la selección de diferentes dosis de intervención de enfermería, según cada actividad en particular.

Investigar sobre la dosis de la intervención de enfermería puede contribuir a que los enfermeros de la práctica clínica tengan claro «qué intervenciones de enfermería proporcionar y en qué cantidad debe proporcionarse cada intervención para alcanzar el resultado deseado»^(5)^, minimizando la variabilidad de la asistencia prestada a los pacientes. Para ello, es igualmente importante incluir debates que aborden las actividades de enfermería integradas en estas intervenciones, ya que las estructuran.

La segunda perspectiva, es decir, la de la gestión del personal de enfermería, se puede encontrar en el término «dosis de enfermería», definido como «el nivel (es decir, el número y el tipo) de personal de enfermería necesario para proporcionar los cuidados que producen los resultados deseados para el paciente»^(6)^. En este contexto, para los autores, la «dosis de enfermería» sería «una variable estructural que captura la capacidad de las enfermeras para proporcionar cuidados de alta calidad en hospitales de cuidados agudos»^(6)^.

A la luz de los autores citados anteriormente, dos atributos reflejan el concepto de dosis de enfermería: ingredientes activos e intensidad. El primero representaría los elementos esenciales y diferenciadores de los enfermeros en relación con otros profesionales de la salud, y tendría como indicadores críticos el conocimiento teórico y práctico de los enfermeros y la combinación de habilidades. La intensidad representaría el potencial para las interacciones entre enfermeros y pacientes, y se operacionalizaría en términos de cantidad y frecuencia^(6)^. La perspectiva desde la que se realizarán las reflexiones en este estudio se aproxima a la comprensión del término «dosis de intervención de enfermería» en relación con la asistencia de enfermería y sus repercusiones^(5)^.

La literatura ha señalado con preocupación la cuestión de la dosis de la intervención de enfermería, «más aún si pensamos en el camino que queda por recorrer en la estructuración de la clínica de enfermería, en el desarrollo de investigaciones clínicas sólidas, el control de la dosis de la intervención de enfermería es crucial. Es necesario demostrar la eficacia y la efectividad de las intervenciones, así como las especificidades de la dosis terapéutica empleada»^(7)^ ante un fenómeno identificado como susceptible de ser tratado por enfermeros.

Ante lo expuesto, se espera que este estudio contribuya a la práctica profesional y ayude a que la toma de decisiones terapéuticas contemple todos los elementos prescriptivos, a saber: fecha de elaboración de la prescripción; verbo que declara la acción a realizar; quién debe realizarla: el sujeto; la frase descriptiva que contemple cómo, cuándo, dónde, con qué frecuencia, durante cuánto tiempo o en qué cantidad; además de la firma del prescriptor^(8)^. Un estudio de revisión señaló la dificultad de discernir la dosis de la intervención en los estudios revisados para respaldar la replicabilidad^(9)^. Discutir desde la perspectiva «¿por cuánto tiempo o en qué cantidad?»^(8)^ puede ayudar a desvelar una toma de decisiones terapéuticas en enfermería más precisa y replicable. Así, se objetivó comprender las repercusiones de la dosis de la intervención de enfermería para la práctica profesional.

## MÉTODO

Se trata de un estudio de reflexión teórica, elaborado a partir de un *corpus* de textos seleccionados por conveniencia que contribuyeron a la comprensión de la problemática planteada, es decir, la comprensión de las repercusiones de la dosis de la intervención de enfermería en la práctica profesional. El ejercicio reflexivo se inició a partir de la siguiente pregunta: ¿cuáles son las repercusiones de la dosis de la intervención de enfermería en la práctica profesional? Se consideró que, en la asistencia diaria, las actividades de enfermería se encuentran en el nivel más bajo de abstracción y mayor concreción, y son parte integrante e indisociable de las intervenciones de enfermería, según el sistema de lenguaje estandarizado adoptado en esta reflexión, es decir, la Clasificación de Intervenciones de Enfermería^(4)^, partiendo de las actividades para comprender las repercusiones de la intervención en lo que respecta a la dosis de la intervención de enfermería. Los diagnósticos de enfermería se consideraron a partir de los diagnósticos de enfermería de NANDA-Internacional^(3)^.

Teniendo en cuenta las cuestiones éticas, el estudio no requiere su presentación ante el Comité de Ética en Investigación, sin embargo, se garantizó el acceso a las fuentes de investigación de forma ética, asegurando la integridad científica a partir de ellas, así como las buenas prácticas para la producción del conocimiento presentado en esta reflexión teórica. Para ello, se ha delineado el tema reflexivo «Repercusiones de la dosis de la intervención de enfermería en la práctica profesional».

### Repercusiones de la Dosis de la Intervención de Enfermería en la Práctica Profesional

Para contextualizar mejor la problemática planteada, se recurre a un caso clínico simulado, en el contexto de la enfermería perioperatoria, teniendo como fenómeno de interés el dolor:

Anciana, 67 años, sometida con urgencia a una cirugía exitosa para tratar una fractura de cuello femoral el 23 de agosto de 2024, alrededor de las 23:00. El 24 de agosto, a primera hora de la mañana, a las 06:00, la paciente comunica al enfermero de guardia que siente dolor en el lugar donde se realizó la intervención quirúrgica, en el momento en que este realiza la ronda de vigilancia clínica de sus pacientes. En ese momento, la paciente fue evaluada mediante la Escala Verbal Numérica (EVN), indicando un nivel de dolor de 8 (0–10). A continuación, el enfermero revisó la prescripción médica e identificó en ella el punto 3: dipirona 1,0 g, intravenosa (IV) cada 6 horas, a las 7:00. Tras tomar las medidas relacionadas con la práctica segura para la administración de medicamentos, se administró el ítem prescrito y, aproximadamente 35 minutos después de recibir la medicación, la paciente ya refería un nivel de dolor de 4 puntos en la EVN, en comparación con la primera evaluación. Después de 60 minutos, refería una leve molestia, que cuantificó en 1 punto en la EVN, en el momento de la reevaluación por parte del enfermero.

A partir del caso clínico simulado, cabe destacar que la dipirona, dependiendo de la edad del cliente, el peso o incluso la vía de administración, tendrá diferentes dosis y tiempos de inicio de acción también diferenciados: para adultos y adolescentes mayores de 15 años (> 31 kg), se consideran dosis entre 500 y 750 mg, por vía oral de 1 a 4 veces al día, y dosis entre 1000 y 2500 mg, por vía intramuscular o intravenosa, de 1 a 4 veces al día. En pediatría (mayores de 3 meses), por ejemplo, la dosis por vía oral en niños de hasta 30 kg debe situarseentre 10 y 15 mg/kg hasta 4 veces al día. La dipirona está indicada para el tratamiento de manifestaciones dolorosas y fiebre^(10)^.

Del párrafo anterior se desprende que la intervención colaborativa de administración de analgésicos^(4)^ se basa en criterios prescriptivos claros (edad, peso del paciente y vía de administración) para el medicamento (tratamiento) en cuestión, es decir, dosis mínimas y máximas, además del intervalo de administración entre dosis, que se han seleccionado a partir de dichos criterios. Partiendo del caso clínico en cuestión, se considera que el enfermero debe buscar, de manera similar, criterios posológicos claros para la asistencia de enfermería, de modo que estos criterios le permitan intervenir de forma autónoma en los fenómenos de especial interés para la enfermería y circunscribir, de forma objetiva, cómo prescribir tratamientos a partir de las intervenciones de enfermería y las dosis que estas pueden abarcar.

A la luz de los conocimientos disciplinarios, base de la práctica autónoma del enfermero, este debe ser capaz de comprender «qué» sabe la enfermería - diagnósticos de enfermería, «cómo» trata ese «qué» (intervenciones de enfermería), así como los posibles resultados clínicos - resultados de enfermería esperados y alcanzables^(1)^. Para influir en dichos resultados, es necesario considerar «cuánto» se implementa del «cómo», a partir de un «qué» determinado: la dosis de la intervención de enfermería. Esta sería la base para influir en los resultados esperados y alcanzables^(1)^, además de modificar los desenlaces clínicos en enfermería de forma autónoma, desvelando las relaciones entre los elementos de la asistencia de enfermería en el plano asistencial de enfermería. También hay que tener en cuenta que las intervenciones de enfermería se relacionan entre sí para modificar los comportamientos de salud. En este sentido, cabe reflexionar: ¿cómo dosificar la información en el contexto de la educación en salud?

Una vez identificado un diagnóstico, se establece un resultado esperado y alcanzable^(1)^ inicial, una «foto inicial»^1^ de la realidad que se va a abordar. Con esta «foto inicial» en mano, el enfermero recurrirá a intervenciones de enfermería para tratar el diagnóstico de enfermería prioritario o «diagnóstico de trabajo»^2^, con el fin de influir en los posibles resultados clínicos. A partir de las reevaluaciones, se obtendrán «fotos parciales» y/o «fotos finales», es decir, las respuestas de los clientes a los tratamientos prescritos y aplicados. El «diagnóstico de trabajo», en esta reflexión, puede entenderse como el fenómeno de enfermería que debe abordarse en un momento determinado del plan de asistencia de enfermería; por ejemplo, a lo largo de 12 o 24 horas de un turno, a pesar de otras posibilidades diagnósticas.

A partir del «diagnóstico de trabajo», el enfermero analizará y establecerá cómo se ha comportado el paciente ante el plan asistencial de enfermería planificado e implementado, lo que permitirá identificar y realizar un seguimiento de los resultados clínicos a corto, medio y largo plazo, base para la evolución de la enfermería. Es importante tener en cuenta que los resultados esperados y alcanzables^(1)^ iniciales, provienen de la respuesta humana identificada que, dependiendo de su tipo, deberán mejorarse, reducirse, mantenerse o recibir otra clasificación que les corresponda. En vista de ello, los resultados iniciales, a partir de un corte temporal evaluativo, adquirirán la connotación de parciales o finales cuando se reevalúen, acción característica de la evolución de la enfermería.

La identificación de «problemas existentes, condiciones de vulnerabilidad o disposiciones para mejorar los comportamientos de salud»^(1)^ será la base para la toma de decisiones terapéuticas en enfermería. Los problemas existentes y/o las condiciones de vulnerabilidad pueden expresar diferentes grados de gravedad, como la extensión de la presentación del fenómeno identificado, lo que desafía a los enfermeros a seleccionar la dosis de la intervención de enfermería según la gravedad expresada por cada cliente, ya que el tratamiento y la dosis seleccionados y prescritos, tomando como referencia el fenómeno identificado, deben tener la capacidad de revertir, estabilizar y/o mantener una determinada condición clínica priorizada: un diagnóstico de enfermería. La perspectiva de un estado para el diagnóstico de enfermería se basa en la literatura de enfermería^(7,11)^.

Además, desde la perspectiva de la gestión de riesgos asistenciales, las intervenciones de enfermería y, en consecuencia, las dosis seleccionadas y prescritas, deben ser capaces de llevar a cabo una gestión adecuada de los riesgos clínicos identificados. Supongamos que un paciente ingresa en una unidad hospitalaria y, basándose en un instrumento de evaluación de riesgos^(12)^, un enfermero identifica que tiene un alto riesgo de caídas, ya que ha obtenido una puntuación de 14 puntos en la evaluación. A continuación, ingresa otro paciente, que ha obtenido una puntuación de 10 puntos y clasificado como de riesgo moderado de caídas, el enfermero responsable de los dos planes de asistencia de enfermería selecciona la intervención de prevención de caídas^(4)^, definida como «imposición de precauciones especiales en pacientes con riesgo de lesiones por caídas»^(4)^. A partir de las puntuaciones obtenidas, se entiende que, aunque representan diagnósticos similares (riesgo de caídas en adultos^(3)^), la gravedad del fenómeno fue diferente, lo que se justifica por las diferentes puntuaciones y, por lo tanto, requerirá dosis de intervención de enfermería que tengan en cuenta «posologías» diferentes.

Ampliando las reflexiones a partir de las situaciones clínicas simuladas anteriores, en el primer caso se trata de un paciente de 70 años con un nivel educativo superior, contable jubilado, pero poco activo y dependiente para el autocuidado. Además, mostró cierta resistencia a las orientaciones y niega el riesgo. En el segundo ejemplo, se trata de otro anciano, ahora de 75 años, activo, que, a pesar de tener un nivel educativo inferior, muestra una mayor adherencia a las orientaciones preventivas. Ambos pacientes tienen un diagnóstico de riesgo de caídas en adultos^(3)^, pero lo expresan clínicamente de manera diferente. A partir de esta provocación, se plantea la siguiente pregunta: ¿qué dosis de intervención de enfermería se debe prescribir en forma de orientación y vigilancia clínica para ambos pacientes, dado que el riesgo se presenta en intensidades o grados diferentes? Aunque el contexto socioambiental adoptado en los ejemplos anteriores es el hospitalario, es posible extrapolar las reflexiones de este texto a otros escenarios, como la Atención Primaria de Salud, ya que es el fenómeno prioritario, así como sus características clínicas, los que serán determinantes para la selección de la dosis de la intervención de enfermería.

Inspirándose en la literatura pertinente^(1,3-7,11)^, se entiende que la dosis de la intervención de enfermería se refiere a la cantidad o intensidad necesarias para que una determinada intervención de enfermería, cuando se implementa, alcance el efecto esperado, de manera que trate a una persona, familia, colectividad o grupos especiales que presenten una determinada respuesta humana, influyendo en resultados esperados y alcanzables en materia de enfermería y salud y modificando o manteniendo los desenlaces clínicos de enfermería por la alteración de su estado —presente, mejorado, empeorado, mantenido o resuelto— de un determinado diagnóstico de enfermería prioritario o de trabajo en un determinado contexto socioambiental. Las intervenciones que contemplen aspectos cuantificables, representados por mililitros (ml), miligramos (mg), centímetros (cm), entre otras medidas, deberán tener su dosis considerada a partir de este tipo de parámetro, expresando de manera objetiva la perspectiva de la dosis de la intervención a prescribir e implementar.

Cuando las intervenciones de enfermería impliquen aspectos conductuales y/o actitudinales, basándose en la interacción entre el enfermero y el cliente, se deduce que la dosis de la intervención de enfermería se entiende a partir de medidas de intensidad y regularidad interactivas, ya que guardan una estrecha relación con el fenómeno de la repetición y/o refuerzo de la oferta de la intervención. Por ejemplo, aquellas intervenciones de enfermería que contemplan orientaciones de educación en salud, ya que la dosis de la intervención tiene que ver con modificaciones o mantenimiento de comportamientos de salud expresados por la persona, la familia, la colectividad o grupos especiales que pueden demandar el refuerzo de esas mismas orientaciones.

El enfoque teórico-práctico del fenómeno de la dosis de la intervención de enfermería, desde esta perspectiva, se reviste de un complejo movimiento guiado por la interacción entre el enfermero y la persona, la familia, la colectividad o los grupos especiales, y encierra en su seno un componente relacional propio que, para ser comprendido, exige un encuentro cara a cara auténtico, en el que ambas partes implicadas tengan voz y espacio para expresarse de forma contributiva. Se comprende el desafío de establecer la dosis de la intervención de enfermería a la luz del fenómeno interactivo de una orientación en salud, ya que el contexto y la cultura de cada persona son ópticas sensibles de interpretación del mundo y de las interacciones que se procesan en él. Por lo tanto, son factores que deben tenerse en cuenta en la selección, prescripción y aplicación de las acciones de enfermería, es decir, en la toma de decisiones terapéuticas en enfermería, que también debe considerar la dosis de la intervención de enfermería.

En este sentido, se descubre una praxis de enfermería relacional y humana única en el contexto de las ciencias de la salud, sin la cual no se puede conocer la dosis ni establecer su posología, que está impregnada, entre otros aspectos, por el respeto mutuo y el reconocimiento de los seres en interacción, en un contexto socioambiental determinado y guiado por la práctica educativa. La intensidad de cada intervención se evaluará *in situ* y en el momento en que el enfermero la ponga en práctica junto a la cama o en cualquier otro escenario, ya que la *retroalimentación*, el nivel de comprensión de una determinada orientación o incluso la capacidad de reproducción a partir de lo que se ha orientado serán obligatorios para comprender qué dosis de la intervención de enfermería debe considerarse en los diversos contextos educativos interactivos.

Al igual que cualquier otro tratamiento, se debe considerar que las intervenciones de enfermería, desde su implementación, pueden tener efectos secundarios que no siempre serán perceptibles «a simple vista», ya que las intervenciones de enfermería implican una importante carga de interacción. Por lo tanto, es necesario debatir y enfatizar este tema hasta sus últimas consecuencias, para que tanto la dosis como los resultados de su implementación se consideren en su totalidad, teniendo en cuenta los efectos secundarios y/o eventos adversos. Una posibilidad de comprender cómo repercuten las intervenciones de enfermería en el contexto interactivo serían las sensaciones de bienestar o malestar resultantes de las acciones puestas en práctica a partir de las prescripciones de enfermería. En este sentido, es imprescindible tener en cuenta la comunicación verbal y no verbal.

La delimitación de un campo de actuación inequívoco como el de la enfermería, que caracterice de manera unánime lo que se defiende como «cuidados de enfermería», puede sucumbir al exceso de prescripciones que valoran las acciones de vigilancia y la rutina asistencial diaria, entendidas a veces como cuidados de enfermería impregnados, en muchos escenarios asistenciales, de aspectos meramente procedimentales. La vigilancia clínica es parte de la asistencia de enfermería, pero las acciones que consideran el tratamiento clínico modificador son obligatorias para delimitar cada vez más el campo de actuación profesional disciplinario. La vigilancia clínica es un componente actitudinal de las intervenciones de enfermería, pero no debe ser la base exclusiva de los tratamientos de enfermería.

Hay que avanzar más allá de verbos como «monitorizar», «acompañar» y «observar», que tienen una connotación de vigilancia clínica estricta, hacia verbos que asuman un carácter prescriptivo/resolutivo, como «realizar», «administrar», «orientar», «implementar», «desbridar», «retirar» y «añadir», que se acercan a cursos de acción y saber hacer autónomos, transponiendo acciones de derivación. En este sentido, las teorías de enfermería pueden ayudar a delimitar el carácter particular de los fenómenos clínicos, como los de enfermería; por lo tanto, además de ayudar a declarar lo que es singular de la enfermería, comprenden, en esa misma delimitación, cómo tratar la particularidad de cómo se recortan esos fenómenos como los de enfermería.

La selección de la dosis de la intervención de enfermería está relacionada con la *experiencia* clínica individual, estando relacionada con la capacidad de comprender la gravedad del diagnóstico de enfermería identificado, el contexto socioambiental en el que se produce y los posibles resultados clínicos esperados a corto, medio y largo plazo, pero también con la propuesta terapéutica a la luz del plan asistencial multiprofesional, ya que, dependiendo del perfil del paciente, ya sea un paciente clínico o quirúrgico, en cuidados de rehabilitación, en cuidados crónicos o incluso en cuidados paliativos, influirán en la posología de la dosis de la intervención de enfermería. La dosis variará según la extensión de la presentación de los fenómenos clínicos identificados.

En esta reflexión, el concepto de intervención se basa en la Clasificación de Intervenciones de Enfermería, es decir, «cualquier **tratamiento** (énfasis nuestro) que, basado en el juicio y el conocimiento clínico, un enfermero pone en práctica para mejorar los resultados del paciente»^(4)^. Los resultados esperados y alcanzables^(1)^ serán mejorados a medida que el tratamiento prescripto e implementado modifique el estado del diagnóstico de enfermería «de trabajo» expresado a partir de modificaciones o mantenimiento de signos y síntomas de importancia clínica para la enfermería. En este sentido es imprescindible comprender que los diagnósticos de enfermería pueden estar presentes, mejorados, empeorados, inalterados y resueltos^(11).^

En la evolución de enfermería, quinta etapa del PE, preguntas del tipo «¿cómo ha respondido el paciente al tratamiento en las últimas 12 o 24 horas?» son orientadoras para procesos evaluativos precisos que permiten corregir rutas en el plan asistencial de enfermería, ante un pronóstico clínico favorable o desfavorable identificado, es decir, la «predicción del curso de una enfermedad y se expresa como la probabilidad de que un evento particular ocurra en el futuro. Las predicciones se basan en grupos definidos de pacientes y el resultado puede ser diferente para cada individuo. Sin embargo, el conocimiento de los pronósticos probables es útil para determinar el mejor tratamiento. Los factores pronósticos son características asociadas con el resultado en pacientes con la enfermedad en cuestión»^(13)^; en el caso de la enfermería, cómo se comportan las respuestas humanas en un contexto y tiempo determinados para evaluar y definir el pronóstico.

Se entiende que, en el ámbito de los cuidados de enfermería, no basta con seleccionar, prescribir y aplicar intervenciones de enfermería, sino que hay que discutir «cuánto» de cada intervención debe «administrarse» y en qué periodo. No es raro que, en las prescripciones de enfermería de muchas instituciones de salud, especialmente en los hospitales, la prescripción de enfermería se lleve a cabo mediante dos elementos principales: una frase en infinitivo, que determina la actividad que se va a realizar (lo que se va a hacer); y un horario (periodicidad o intervalo de realización de una determinada acción). Sin embargo, los enfermeros y sus equipos deben orientar sus acciones a partir de preguntas como «¿cuánto cuidado de enfermería se debe dispensar y con qué intensidad?».

Por actividades de enfermería se entiende que están «en el nivel concreto de acción, ya que son las conductas o acciones específicas que se toman para implementar una intervención y que ayudan a los pacientes a progresar hacia el resultado esperado»^(4)^. Las actividades que componen cada intervención pueden considerarse pasos que, puestos en práctica, proporcionan una vía para implementar una determinada intervención. Aunque la literatura señala que las investigaciones podrían utilizar las actividades como predictores de la dosis de la intervención, se entiende que contabilizar un número determinado de actividades como base para definir una dosis determinada no contempla la comprensión de la dosis de enfermería que se discute en esta reflexión.

El Cuadro 1 presenta la intervención de enfermería «Prevención de lesiones por presión»^(4)^ y las actividades que la estructuran, base para el ejercicio reflexivo sobre la dosis de la intervención de enfermería que se propone en este artículo. En cada actividad, se buscó comprender las dosis potenciales que deben considerarse o no. En algunas actividades, por la forma en que están descritas, se contemplan más de una actividad en su texto, lo que dificultaría la selección precisa de una dosis. Se entiende también que la Clasificación de Intervenciones de Enfermería no tiene este tipo de compromiso, es decir, un nivel excesivo de descripción que, en opinión de los autores de esta reflexión, no sería el objetivo de la clasificación. Sin embargo, el ejercicio reflexivo realizado ayuda a desvelar la problemática que se quiere discutir.

**Cuadro 1:** Reflexiones teóricas basadas en una intervención de enfermería – Niterói, RJ, Brasil, 2024.

Intervención – Prevención de las lesiones por presión Definición: prevención de las lesiones por presión en una persona con alto riesgo de desarrollarlas.	
**Actividades**	**Ejercicio crítico-reflexivo**
1. Utilizar una herramienta reconocida de evaluación de riesgos para controlar los factores de riesgo de la persona (por ejemplo, la escala de Braden).	1. Las herramientas de evaluación del riesgo ayudan a comprender la gravedad de los riesgos y a definir la intensidad de la prevención que debe prescribirse o aplicarse. En este caso, la dosis estaría relacionada con el intervalo de evaluación y la vigilancia clínica aplicada.
2. Utilizar métodos de medición de la temperatura de la piel para determinar el riesgo de lesiones por presión, de acuerdo con el protocolo de la institución.	2. Los métodos de medición ayudan a comprender la gravedad del riesgo y a definir la intensidad de la prevención que debe prescribirse. En este sentido, el intervalo de evaluación apoyaría la selección de la dosis o de los recursos preventivos a seleccionar.
3. Anime a la persona a no fumar y a evitar el alcohol.	3. Esta actividad se refiere a las orientaciones para dejar de fumar y el consumo de alcohol. Implican el cumplimiento de las medidas de orientación y deben tener en cuenta el nivel de conocimiento y compromiso para dejar de fumar. La dosis en este caso está relacionada con la cantidad/intensidad de información que debe compartirse, así como con el número de enfoques considerados. La comprensión y la modificación del comportamiento a partir de la primera consulta de enfermería serían la base para seleccionar la dosis, que se volvería a evaluar en otras reuniones, ya que es necesario considerar hasta qué punto el individuo está dispuesto a modificar los comportamientos de riesgo.
4. Documentar cualquier incidencia previa de formación de lesiones por presión.	4. La documentación forma parte y es el resultado de la evaluación inicial y continua, y permite comprender cómo se llevó a cabo el tratamiento de enfermería en un momento anterior. No se trata aquí de considerar la dosis de la intervención enfermera, sino la calidad de la evaluación inicial y de los registros profesionales resultantes.
5. Documentar el peso y los cambios de peso.	5. Los cambios en el peso pueden orientar la intensidad de las medidas preventivas y, en consecuencia, la dosis de intervención de enfermería, ya que en función del grado de riesgo deben considerarse una serie de tecnologías y actuaciones de enfermería.
6. Documentar el estado de la piel al ingreso y diariamente.	6. La evaluación inicial de la piel y su respuesta al tratamiento prescrito pueden ayudar a programar y seleccionar las medidas preventivas y los tratamientos. De este modo, existe una correlación directa entre el estado actual de la piel, su riesgo clínico potencial para una lesión determinada y los resultados clínicos esperados. Sin embargo, la actividad concierne al registro profesional.
7. Vigilar intensamente las zonas enrojecidas.	7. La monitorización está relacionada con la vigilancia clínica de enfermería y debe entenderse, en esta realidad, como una dosis, relacionada con la intensidad con la que se realiza la monitorización, directamente relacionada con el intervalo de tiempo y el tiempo dedicado a la evaluación.
8. Eliminar el exceso de humedad de la piel resultante de la transpiración, el drenaje de heridas y la incontinencia fecal y urinaria.	8. Son varios los factores que contribuyen al exceso de humedad, lo que dificulta el establecimiento de una dosis teniendo en cuenta la actividad enfermera en cuestión, ya que la exposición a la humedad puede producirse en diferentes momentos y por diferentes causas. La dosis relacionada con la monitorización continua o la individualización de cada problema/acción/dosis debe discutirse en la prescripción.
9. Aplique barreras protectoras, como cremas o materiales que absorban la humedad, para eliminar el exceso de humedad según proceda.	9. Además de la eliminación de la humedad, se plantea la cuestión de la cantidad de crema barrera que debe aplicarse, ya que esto está relacionado con la dosis de la intervención de enfermería. Además, en la actividad también se mencionan los materiales absorbentes que tendrían otra perspectiva de dosis, vinculada a su capacidad de absorción y, en consecuencia, a las horas de protección que puede ofrecer la tecnología en cuestión por su capacidad para retener un volumen determinado de orina.
10. Cambiar de posición cada 1 o 2 horas.	10. El reposicionamiento es una de las actividades que matiza sensiblemente la comprensión de cuál es la dosis de la intervención enfermera, ya que se identifica que la dosis está ligada al intervalo en el que se produce, es decir, cada 1 ó 2 horas se dispensará una “dosis de cambio de decúbito” para prevenir la aparición de lesiones por presión que, entre otros factores, están relacionadas con la isquemia local que pueda estar experimentando un determinado tejido.
11. Cambie la posición del paciente con cuidado (evite la deformación por fuerzas tangenciales) para evitar lesiones en la piel frágil.	11. Esta actividad indica la forma en que se hacen las cosas y está relacionada con la actividad “Cambiar de posición cada 1 ó 2 horas”; por lo tanto, no sería apropiado hablar de la dosis de la intervención de enfermería en este ítem, sino de la calidad con que se lleva a cabo una determinada acción de cuidado.
12. Coloque el guión de cambio de posición junto a la cama, según proceda.	12. La indicación en el guión sirve como base para que el paciente no repita la posición antes del tiempo indicado; por lo tanto, no es apropiado discutir la dosis de la intervención de enfermería en este ítem, ya que la estrategia serviría a los profesionales como guía para la actividad.
13. Inspeccione la piel sobre las prominencias óseas y otros puntos de presión al menos una vez al día durante el reposicionamiento.	13. La monitorización tiene que ver con la intensidad de un riesgo potencial identificado en un paciente; por lo tanto, en función del nivel de riesgo, la dosis de monitorización debe reajustarse en función de la evaluación de la piel en cada reevaluación, es decir, cuánta monitorización debe prescribirse. En el caso de esta actividad, la relación entre acción y dosis tiene que ver con el intervalo de tiempo en el que se realizan las acciones.
14. Evite masajear sobre prominencias óseas.	14. Guía de buena práctica clínica que complementa la forma de intervenir. No es necesario discutir la dosis de la intervención de enfermería.
15. Colóquelo con almohadas para levantar los puntos de presión de la cama.	15. Guía de buena práctica clínica que complementa la forma de intervenir. No es necesario discutir la dosis de la intervención de enfermería.
16. Mantenga las sábanas limpias, secas y sin arrugas.	16. Guía de buena práctica clínica que complementa la forma de intervenir. No es necesario discutir la dosis de la intervención de enfermería.
17. Haz la cama con pliegues para los dedos de los pies.	17. Guía de buena práctica clínica que complementa la forma de intervenir. No es necesario discutir la dosis de la intervención de enfermería.
18. Utilizar camas y colchones especializados, según proceda.	18. El uso de camas y superficies especializadas debe seleccionarse en función del grado o puntuación de riesgo identificado a partir de una determinada herramienta de evaluación clínica y, en este sentido, estas camas y colchones especializados se seleccionarán en función de su gravedad. Esto está relacionado con la “cantidad” de tecnologías disponibles.
19. Utilizar equipos de cabecera que protejan al paciente.	19. Guía de buena práctica clínica que complementa la forma de intervenir. No es necesario discutir la dosis de la intervención de enfermería.
20. Evite los equipos de tipo “rosquilla” en la zona sacra.	20. Guía de buena práctica clínica que complementa la forma de intervenir. No es necesario discutir la dosis de la intervención de enfermería.
21. Hidratar la piel seca aún intacta.	21. La hidratación está relacionada con la aparición de lesiones por presión, por lo que se debe discutir la dosis de hidratación a prescribir. Las características del producto seleccionado serán obligatorias para seleccionar la dosis de la intervención de enfermería.
22. Evite el agua caliente y utilice jabón neutro al bañarse.	22. Guía de buena práctica clínica que complementa la forma de intervenir. No es necesario discutir la dosis de la intervención de enfermería.
23. Vigilar las fuentes de presión y fricción.	23. La dosis de vigilancia estará relacionada con la gravedad del riesgo de lesiones por presión. Un intervalo entre monitorizaciones puede relacionarse con la dosis en el sentido de cuánta monitorización se aplica.
24. Aplique protectores de talón y codo según proceda.	24. La aplicación de protectores tiene que ver con el grado de riesgo, ya que en función del tipo de riesgo se selecciona una tecnología concreta que sea capaz de proteger al paciente evaluado y su piel. Por ello, es importante seguir las indicaciones del fabricante. En el caso de cremas y *sprays*, la cantidad dispensada durante la aplicación debe considerarse la dosis de la intervención de enfermería. Es importante destacar que la selección de la cantidad de barrera o crema protectora tiene que ver con la extensión de la superficie en riesgo.
25. Facilitar pequeños cambios frecuentes en la posición del paciente.	25. El reposicionamiento menor se considerará a la luz del riesgo de lesión por presión identificado; por lo tanto, la dosis está relacionada con los intervalos de cambio y el grado de riesgo. En este entendimiento, la dosis de la intervención de enfermería y el intervalo de cambio de posición son inseparables.
26. Proporcione un trapecio para ayudar con los cambios frecuentes de posición del paciente.	26. Guía de buena práctica clínica que complementa la forma de intervenir. No es necesario discutir la dosis de la intervención de enfermería.
27. Controlar la movilidad y la actividad de la persona.	27. Los niveles de movilidad y actividad serán determinantes para la intensidad de la aplicación de las medidas preventivas y, por lo tanto, orientarán la definición de la dosis de intervención de enfermería con respecto al reposicionamiento del paciente.
28. Garantizar una ingesta alimentaria adecuada, especialmente de proteínas, vitaminas B y C, hierro y calorías, utilizando suplementos según proceda.	28. Se trata de una actividad colaborativa que, para ser implementada, debe ser discutida con nutricionistas y nutricionistas. En este sentido, la dosis de la intervención debe ser discutida conjuntamente, pero supervisada de cerca por la enfermera y el equipo de enfermería, ya que son los profesionales que comúnmente administran u ofrecen alimentos a los pacientes.
29. Ayudar a las personas a mantener un peso saludable.	29. Mantener un peso saludable es una actividad compleja que engloba una serie de actividades, y la monitorización del peso es una medida que se puede implementar de forma rápida e independiente, pero no debería ser la única. Las enfermeras deben trabajar conjuntamente a través de un plan terapéutico multiprofesional para ayudar al individuo a mantener el peso adecuado a las variables definidas en la evidencia científica. La dosis de esta actividad está relacionada con la cantidad de orientación que se daría para que un individuo pierda peso.
30. Educar a la familia o al cuidador sobre los signos de lesiones cutáneas, según proceda.	30. Esta actividad está relacionada con el aprendizaje y el compromiso de comportamientos de prevención de lesiones por presión establecidos por todos los implicados. La dosificación de la intervención está relacionada con la cantidad de información compartida, la intensidad del enfoque y el intervalo entre orientaciones, es decir, el intervalo entre las dosis de información ofrecidas.

Fuente: Adaptado de Classificação das Intervenções de Enfermagem^(4)^.

El ejercicio reflexivo anterior puso de manifiesto la compleja pero necesaria discusión de la dosis de intervención enfermera a nivel de actividades o acciones concretas, ya que éstas serán prescritas e implementadas en los escenarios de cuidados por la enfermera y el equipo, y orientarán las acciones asistenciales que representan a través de la prescripción enfermera. En este sentido, señalar líneas de actuación es incuestionable, como también lo es describir con claridad el grado en que estas acciones deben llevarse a cabo, el cuánto del cómo. Los tratamientos deben contemplar necesariamente las dosis a prescribir.

En el contexto de los cuidados de enfermería, los familiares y personas significativas son importantes herramientas terapéuticas que los pacientes tienen a su disposición para continuar el tratamiento o incluso prevenir complicaciones de salud, y se convierten en colaboradores, piezas fundamentales que deben ser incorporadas a la hora de seleccionar e implementar las acciones de salud y enfermería. Así, es fundamental evaluar críticamente como los familiares y personas significativas entienden el plan de asistencia de enfermería, ya que deben ser tenidos en cuenta a la hora de seleccionar las intervenciones de enfermería y sus respectivas dosis.

La limitación de esta investigación reside en el contexto de su abordaje, es decir, una reflexión teórica, ya que la complejidad del problema aquí tratado podría ser considerada utilizando métodos más robustos. La investigación respondió al objetivo cuando, a través del ejercicio reflexivo propuesto, identificó las posibles repercusiones de la dosis de intervención enfermera para la práctica profesional.

### Consideraciones Finales

Para comprender la dosificación de la intervención enfermera y sus repercusiones en la práctica profesional, es necesario considerar que su prescripción debe basarse en criterios posológicos claros. Sin estos criterios, es difícil determinar la dosis de la intervención y/o medir el impacto de los tratamientos prescritos por las enfermeras en los resultados clínicos de los pacientes. La perspectiva de la dosis de la intervención enfermera presentada en este texto está delimitada por la Clasificación de las Intervenciones de Enfermería. En esencia, un conjunto de actividades constituye una intervención enfermera. Comprender cómo cada actividad puede requerir una dosis específica, y cómo el conjunto integrado de actividades expresa la dosis de una intervención en su totalidad, es fundamental para entender sus repercusiones en la práctica profesional enfermera.

A la luz del ejercicio de reflexión realizado y del *corpus* textual seleccionado, se esbozan las siguientes repercusiones la posibilidad de medir la eficacia y la efectividad de los tratamientos prescritos por las enfermeras; la reducción de la variabilidad de las acciones prescritas, ya que será posible no sólo elegir un curso de acción, sino también determinarlo, teniendo en cuenta parámetros como la frecuencia, la intensidad y la cantidad; una mayor claridad para los auxiliares y técnicos de enfermería sobre las normas de práctica establecidas por las enfermeras; la construcción de una base de pruebas más sólida para que las enfermeras, en diversos contextos, se sientan seguras al asumir la responsabilidad de los resultados clínicos en enfermería; una mayor replicabilidad de las intervenciones/actividades en los estudios de intervención de enfermería.

Además, incluyen una mayor claridad en la delimitación de las acciones autónomas de las enfermeras como parte del equipo sanitario y un mayor potencial de replicabilidad de los tratamientos prescritos en situaciones similares, lo que puede contribuir a la adquisición de *experiencia* clínica en los procesos de toma de decisiones diagnósticas o terapéuticas. Por último, existe una mayor claridad para futuras actualizaciones de la Clasificación de Intervenciones de Enfermería y una mejor orientación sobre cómo prescribir la asistencia en forma eficaz, repercutiendo en los resultados de esperados y alcanzables y en el estado de los diagnósticos priorizados en el contexto de la práctica clínica.

## Data Availability

Todos los datos que respaldan los resultados de este estudio han sido publicados en el propio artículo.

## Footnotes

1 La asociación de resultados esperados de enfermería a registro fotográficos o fotos «iniciales», «parciales» y «finales» se refiere a la acción de fotografiar heridas o lesiones para que se tengan un parámetro inicial y a partir de este parámetro, se pueda establecer la evolución de ellas en un determinado contexto asistencial, de acuerdo a un período establecido, permitiendo así evaluar los resultados esperados y los alcanzados.

2 La expresión «diagnóstico de trabajo» fue escuchada por primera vez cuando el primer autor de esta reflexión conversaba con la profesora Dra. Maria Marcia Bachion, en un evento conmemorativo del aniversario del *Grupo de Estudos em Sistematização da Assistência de Enfermagem* de la *Universidade Federal Fluminense* (GESAE-UFF), celebrado en Río de Janeiro.
